# Cleavage Factor I Links Transcription Termination to DNA Damage Response and Genome Integrity Maintenance in *Saccharomyces cerevisiae*

**DOI:** 10.1371/journal.pgen.1004203

**Published:** 2014-03-06

**Authors:** Hélène Gaillard, Andrés Aguilera

**Affiliations:** Centro Andaluz de Biología Molecular y Medicina Regenerativa CABIMER, Universidad de Sevilla-CSIC, Sevilla, Spain; Duke University, United States of America

## Abstract

During transcription, the nascent pre-mRNA undergoes a series of processing steps before being exported to the cytoplasm. The 3′-end processing machinery involves different proteins, this function being crucial to cell growth and viability in eukaryotes. Here, we found that the *rna14-1*, *rna15-1*, and *hrp1-5* alleles of the cleavage factor I (CFI) cause sensitivity to UV-light in the absence of global genome repair in *Saccharomyces cerevisiae*. Unexpectedly, CFI mutants were proficient in UV-lesion repair in a transcribed gene. DNA damage checkpoint activation and RNA polymerase II (RNAPII) degradation in response to UV were delayed in CFI-deficient cells, indicating that CFI participates in the DNA damage response (DDR). This is further sustained by the synthetic growth defects observed between *rna14-1* and mutants of different repair pathways. Additionally, we found that *rna14-1* suffers severe replication progression defects and that a functional G1/S checkpoint becomes essential in avoiding genetic instability in those cells. Thus, CFI function is required to maintain genome integrity and to prevent replication hindrance. These findings reveal a new function for CFI in the DDR and underscore the importance of coordinating transcription termination with replication in the maintenance of genomic stability.

## Author Summary

DNA damage occurs constantly in living cells and needs to be recognized and repaired to avoid mutations. DNA repair is particularly relevant for lesions occurring in actively transcribed DNA strands because the RNA polymerase cannot proceed through a damaged site. Stalled RNA polymerases and persisting DNA lesions can lead to genome instability or cell death. Specific mechanisms to repair obstructing DNA lesions are found from bacteria to higher eukaryotes, their malfunction leading to severe genetic syndromes in humans. Termination of transcription comprises cleavage and polyadenylation of the nascent transcript and displacement of the RNA polymerase from its DNA template. These processes, which are crucial for cell viability and growth in eukaryotes, require two major multi-subunit complexes in budding yeast. Here, we found that one of these complexes, Cleavage Factor I (CFI), participates in the cellular response to DNA damage. In addition, we found that CFI dysfunction leads to replication defects, conceivably mediated by stalled RNA polymerases, rendering cell cycle checkpoints mandatory to prevent genomic instability. Our findings emphasize the importance of coordinating transcription termination, DNA damage response and replication in the maintenance of genomic stability suggesting that CFI plays a fundamental function in the coupling of these processes.

## Introduction

All cells are continuously exposed to DNA damaging agents, which can arise from exogenous sources or from endogenous metabolic processes. The DNA damage response (DDR) includes the activation of checkpoints and induction of DNA repair pathways. DNA lesions can generate structural distortions that interfere with basic cellular functions like transcription and replication. Such helix-distorting DNA lesions are generally handled by nucleotide excision repair (NER), which can be divided into global genome repair (GG-NER) and transcription-coupled repair (TC-NER) sub-pathways, depending on whether the DNA lesion is located anywhere in the genome or on the transcribed strand (TS) of an active gene, respectively. At transcribed genes, TC-NER acts when elongating RNA polymerase (RNAP) stalls at bulky DNA lesions such as UV-induced cyclobutane pyrimidine dimers (CPDs) (reviewed in [1], [2]). Transcription down-regulation and proteasome-mediated degradation of engaged RNAPII take place as part of the DDR to UV-induced damages [3], [4]. In humans, defects in TC-NER are responsible for two severe genetic disorders called Cockayne Syndrome (CS) and UV Sensitivity Syndrome (reviewed in [5], [6]). In *S. cerevisiae*, the major TC-NER factor is Rad26, the yeast homologue of CS protein B (CSB) [7]. However, residual TC-NER activity remains in the absence of Rad26, indicating that other factors are also involved in the process [7], [8]. Mutations in several transcription and messenger ribonucleoprotein (mRNP) biogenesis factors including the RNAPII subunit Rpb9, THO, THSC/TREX-2, Paf1, and Ccr4-NOT are partially defective in TC-NER in yeast [9]–[12].

During the past few years it has become clear that the different mRNA processing steps (including 5′-end capping, splicing, and 3′-end cleavage), mRNP export, and transcription are connected to each other (reviewed in [13]) and that surveillance mechanisms ensure that these processes occur in a coordinated manner (reviewed in [14]). THO and THSC/TREX-2 both work at the interface between transcription elongation, mRNP biogenesis and export and defects are characterized by a strong transcription-dependent hyperrecombination phenotype (reviewed in [15], [16]). THO might also act in the process of transcription termination, as *in vitro* assays suggest that THO mutants lead to polyadenylation defects [17]. Interestingly, other factors required for proficient TC-NER also function during transcription termination. The Paf1 transcription elongation factor contributes to the recruitment of 3′-end processing factors necessary for accurate transcription termination (reviewed in [18]). The Ccr4-NOT complex acts, among other gene expression functions, during transcription elongation and interacts with mRNP export factors (reviewed in [19]).

In the yeast *Saccharomyces cerevisiae*, the transcription termination machinery can be divided into three different sub-complexes: cleavage factor IA (CFIA), cleavage factor IB (CFIB), and cleavage and polyadenylation factor (CPF). CFIA is comprised of the Rna14, Rna15, Pcf11, and Clp1 proteins. CFIB consists of the RNA-binding protein Hrp1, which is tightly associated with CFIA. The CPF complex is a large complex that can be further classified into the cleavage factor II (CFII) made out of the Cft1, Yhh1, Pta1, Brr5, Ysh1, Cft2, and Ydh1 proteins; the polyadenylation factor I made of Fip1, Yth1, and Psf1; and other proteins including the Pap1 polymerase. *In vitro* reconstitution of the cleavage reaction demonstrated that it requires the joint action of CFIA, CFIB, and CFII [20], [21], while additional proteins such as the 5′-3′-exoribonuclease Rat1 are required for termination downstream of poly(A) sites *in vivo* and dismantling of RNAPII complexes *in vitro* [22]–[24]. In addition to their role in cleavage, many of the components of the cleavage machinery are required for transcription termination downstream of the poly(A) site and polyadenylation of the transcript (reviewed in [25], [26]). Notably, the CFIA *rna14-1* and *rna15-1* mutants suffer from transcription elongation defects and increase in transcription-dependent hyper-recombination [27], suggesting that the CFIA complex serves important functions in transcription beyond termination and 3′-end processing.

To assess the possible function of RNA 3′-processing and transcription termination on TC-NER, we analysed the impact of a number of mutations on the DDR and the repair of UV-induced lesions. We found that CFI mutants become sensitive to UV in the absence of GG-NER, but surprisingly are proficient for CPD repair. By contrast, DDR is compromised in those cells, as seen by RNAPII degradation and checkpoint activation analyses upon UV irradiation. In addition, we show that *rna14-1* cells are impaired in cell cycle progression and rely on a functional G1/S checkpoint to prevent genomic instability and cell death. Our study reveals that CFI functions in DDR and is required for genomic integrity maintenance in yeast.

## Results

### CFI mutants are UV-sensitive in the absence of global genome repair

**Figure 1:**
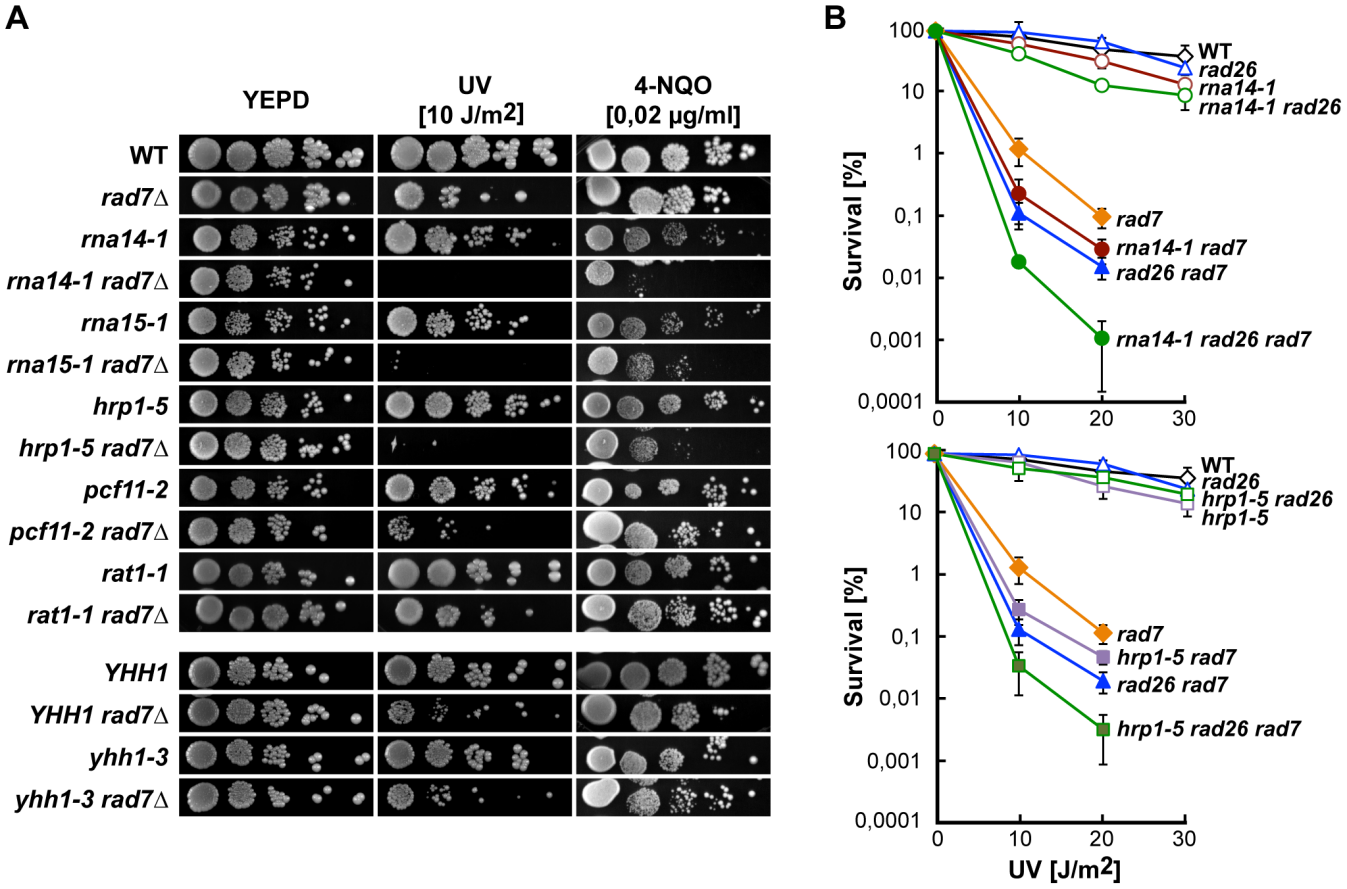
CFI mutations lead to UV sensitivity in the absence of global genome repair. (A) UV and 4-NQO sensitivity of six different transcription termination mutants alone and in combination with the *rad7Δ* mutation. (B) UV sensitivity curves of strains carrying single, double and triple combinations of *rna14-1* (top) and *hrp1-5* (bottom) together with *rad7Δ* and *rad26Δ* mutations. Average values from at least three independent experiments and corresponding standard deviations are plotted.

We first analysed the sensitivity of several transcription termination mutants to DNA damage in the absence of Rad7, a protein required for GG-NER in yeast. Growth of each double mutant was compared to the growth of *rad7Δ* after irradiation with UV light and in the presence of the UV-mimetic agent 4-nitroquinoline 1-oxide (4-NQO) (Figure 1A). The *rna14-1 rad7Δ*, *rna15-1 rad7Δ*, and *hrp1-5 rad7Δ* double mutants were significantly more affected by UV irradiation or 4-NQO than the respective single mutants, while the remaining assayed alleles (*pcf11-2, rat1-1, and yhh1-3*) were not. Notably, deletion of the *RAD26* gene, which encodes the main TC-NER factor, further increased the sensitivity of *rna14-1 rad7Δ* and *hrp1-5 rad7Δ* mutants, indicating that the *rna14-1* and *hrp1-5* alleles are not epistatic to *rad26Δ* (Figure 1B).

**Figure 2:**
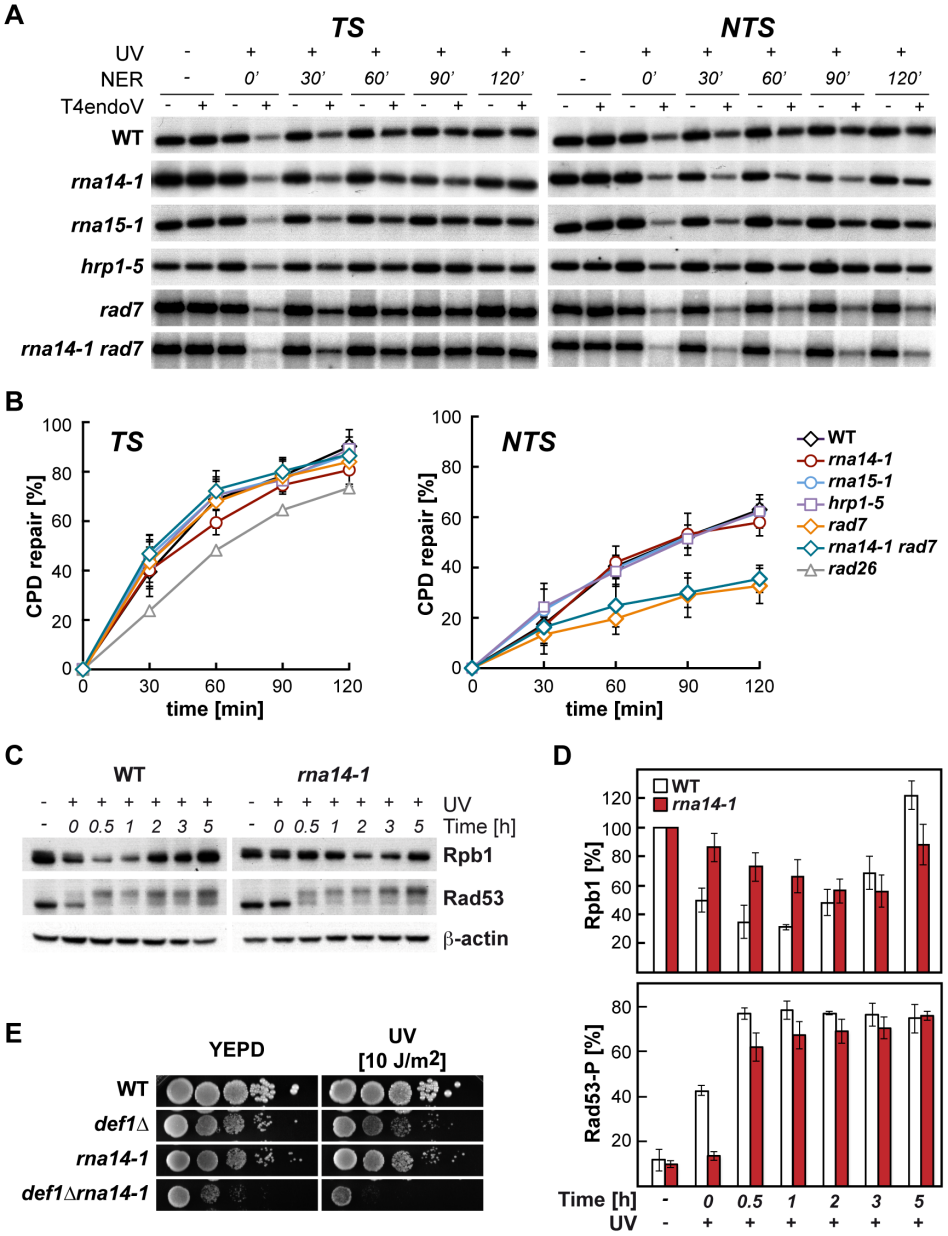
Normal CPD repair and DNA-damage response alteration in transcription termination mutants. (A) Southern analysis showing repair of a 4.4-kb (*Nsi*I/*Pvu*I) *RPB2* fragment in wild-type, *rna14-1, rna15-1*, *hrp1-5, rad7Δ and rna14-1 rad7Δ* cells. Initial damage was on the average 0.247±0.091 CPD/Kb in the transcribed strand (TS, left) and 0.245±0.098 CPD/Kb in the non-transcribed strand (NTS, right). The remaining intact restriction fragment after treatment with T4endoV (+UV, +T4endoV) corresponds to the fraction of undamaged DNA. Non-irradiated DNA (-UV) and DNA not treated with T4endoV (-T4endoV) were used as controls. (B) Graphical representation of the quantified results. The CPD content was calculated using the Poisson expression, -ln (RF_a_/RF_b_), where RF_a_ and RF_b_ represent the intact restriction fragment signal intensities of the T4endoV- and mock-treated DNA, respectively. Repair curves were calculated as the fraction of CPDs removed *vs*. repair time. Average values derived from three independent experiments are plotted with their standard deviation. Repair curve of *rad26* (data taken from [9]) is depicted for the TS. (C) Western analysis of Rpb1 and Rad53 upon UV irradiation in *rna14-1* and wild-type cells. β-actin is shown as loading control. (D) Graphical representation of the quantified results from Rpb1 and Rad53 Western analyses. The amount of Rpb1 is shown as the percentage of Rpb1 in the non-irradiated sample. The percentage of hyper-phosphorylated Rad53 is plotted for each condition. Average values derived from three independent experiments are plotted with their standard deviation. (E) Genetic interaction analysis between the *rna14-1* and the *def1Δ* mutants. Serial dilutions (10-fold) of exponentially growing cultures are shown. This panel complemented with the data of the *rna15-1 def1Δ*, *hrp1-5 def1Δ*, and *rat1-1 def1Δ* mutants are shown in Figure S2.

Because UV sensitivity in the absence of GG-NER is a phenotype mostly associated with TC-NER deficiencies, we tested whether functional CFI was required for proficient TC-NER by monitoring the repair rates of the transcribed (TS) and non-transcribed (NTS) strands of the constitutively expressed *RPB2* gene in *rna14-1, rna15-1*, and *hrp1-5* cells (Figure 2, A and B). With the exception of the 60 min. time-point in *rna14-1*, which is seemingly lower than the wild type on the TS, no significant differences were observed between the repair rates of the mutants and the wild type in either *RPB2* strand. Repair experiments were thus performed in *rad7Δ* and *rna14-1 rad7Δ* cells. As can be seen in Figure 2 (A and B), both strains show a similar low repair on the NTS and are repair-proficient on the TS. Together, our results indicate that the *rna14-1, rna15-1*, and *hrp1-5* mutants are repair-proficient for CPDs. Because deficiencies in NER may cause an increase in recombinational repair and *rna14-1* cells show moderate hyper-recombination [27], we assessed whether recombination increased upon UV irradiation in *rna14-1*, *rad7Δ*, and *rna14-1 rad7Δ* cells. For this, we used a direct-repeat (LY) and an inverted-repeat (SU) plasmid-based system [28]. As expected, *rad7Δ* cells show an increase in recombination upon UV-damage in both systems (13- and 35-fold, Figure S1). However, recombination frequencies did not increase upon UV irradiation in *rna14-1* cells, suggesting that UV damage is efficiently repaired by NER. Notably, the *rna14-1 rad7Δ* double mutant shows UV-dependent increase in recombination frequency as compared to the *rad7Δ* mutants in the direct-repeat system -but not in the inverted-repeat system- suggesting that these cells suffer from increased genomic instability that is not linked to increased CPD repair deficiencies.

### The DNA damage response is delayed in *rna14-1* mutants

The cellular response to UV-induced damage involves, in addition to checkpoint activation, proteosomal degradation of RNAPII [3]. To check the functionality of the DDR in *rna14-1* cells, we analysed the stability of Rpb1, the largest subunit of RNAPII, and activation of the Rad53 checkpoint protein upon UV irradiation by Western analysis (Figure 2, C and D). Interestingly, UV-induced Rpb1 degradation was less pronounced and severely delayed in *rna14-1* cells as compared to the wild type. Activation of the DNA-damage checkpoint, monitored by the appearance of hyper-phosphorylated Rad53 upon UV irradiation was delayed in *rna14-1* cells as compared to the wild type, in which Rad53 phosphorylation occurs immediately upon UV irradiation. In addition, the *rna14-1* mutation did not increase the sensitivity to UV or 4-NQO of cells lacking either one of the DNA-damage checkpoint proteins Rad9 and Mec1 (Figure S2), suggesting that CFI might act within the canonical checkpoint pathways. To gain more insights into the function of CFI in the cellular response to UV-induced damage, Rpb1 stability and Rad53 phosphorylation were also analysed in cells bearing the *rna15-1*, *hrp1-5* and *pcf11-2* mutations (Figure S3). Both *rna15-1* and *pcf11-2* cells were partially impaired in UV-induced Rpb1 degradation while *hrp1-5* cells behaved similarly to the wild type. However, Rad53 phosphorylation was delayed in the *rna15-1* and *hrp1-5* mutants but not in *pcf11-2* cells. These interesting results suggest that UV-induced Rpb1 degradation might not depend on Rad53 activation. Previously, deletion of the *DEF1* gene was shown to increase the sensitivity to UV in the absence of GG-NER without affecting DNA repair at the molecular level and to impair UV-dependent Rpb1 degradation [29]. Thus, we assayed viability and sensitivity of *rna14-1 def1Δ, rna15-1 def1Δ, hrp1-5 def1Δ, and rat1-1 def1Δ* double mutants to assess possible genetic interactions and observed strong synthetic sickness even in the absence of exogenous damage in all strains except *hrp1-5 def1Δ* (Figures 2E and S4). These interesting genetic interactions suggest that Def1 and CFI might have complementary functions for cell growth, which eventually rely on alternative ways to regulate RNAPII turnover. Although the penetrance of the different alleles differs depending on the analysed phenotype, our data indicate that CFI is required for the cellular response to UV-induced damage.

### The ability to withstand DNA damage is reduced in CFI mutants

**Figure 3:**
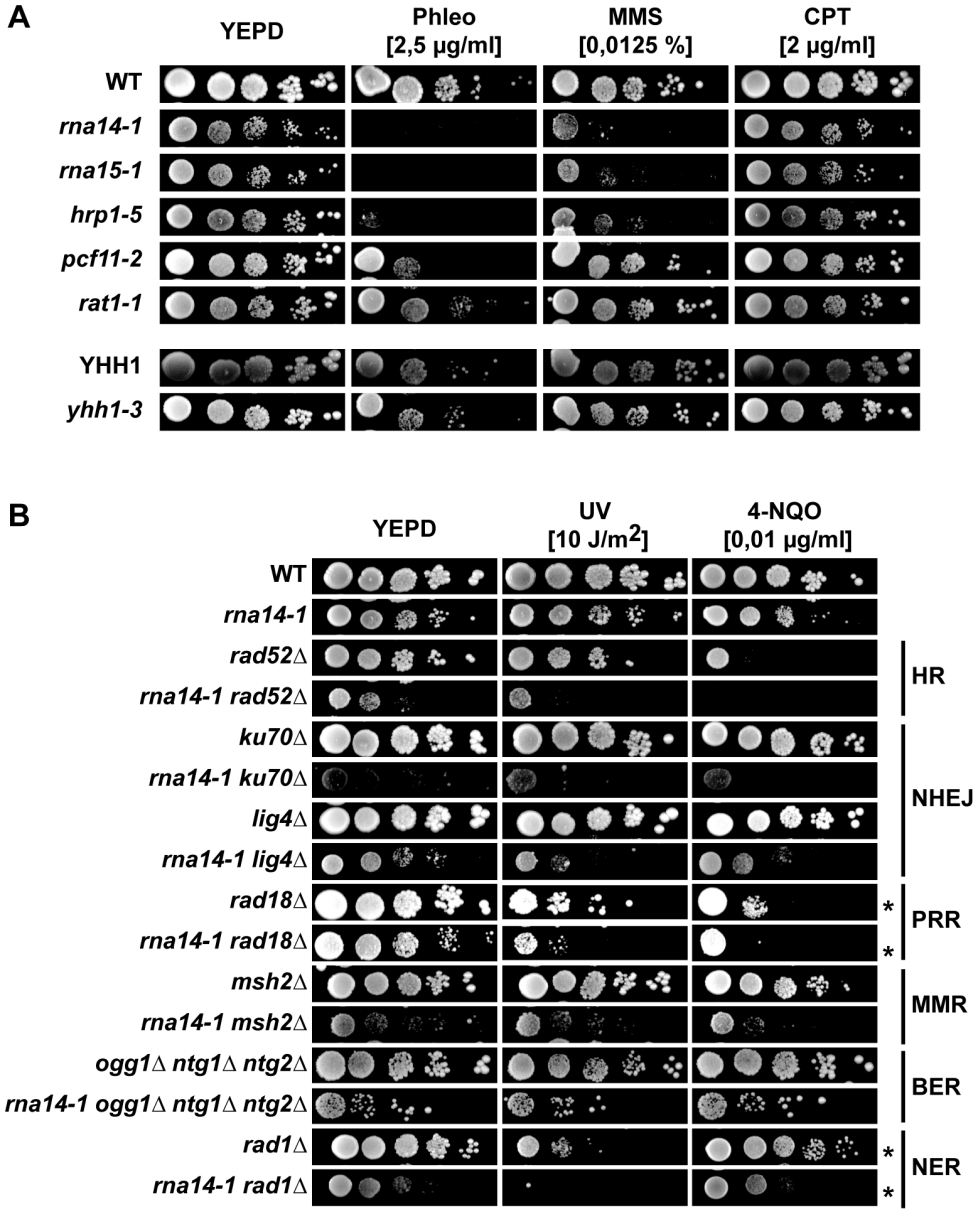
Transcription termination mutants do not tolerate compromised DNA repair. (A) Sensitivity to Phleomycin (Phleo), methyl methanesulfonate (MMS), and camptothecin (CPT) of six transcription-termination mutants. 10-fold serial dilutions of exponentially growing cultures are shown. (B) Analysis of genetic interactions between *rna14-1* and mutants impaired in homologous recombination (HR), non-homologous end joining (NHEJ), post-replicative repair (PPR), mismatch repair (MMR), base excision repair (BER), and nucleotide excision repair (NER). 10-fold serial dilutions of exponentially growing cultures are shown. * indicates that the UV dose was 2 J/m^2^.

Sensitivity analysis of different termination mutants to distinct DNA damaging agents revealed that the *rna14-1*, *rna15-1*, and *hrp1-5* mutants were sensitive to Phleomycin and to methyl methansulfonate (MMS) in contrast to the *pcf11-2*, *rat1-1*, and *yhh1-3* cells, which were either slightly or not sensitive to those genotoxic agents (Figure 3A). Interestingly, the three alleles conferring significant sensitivity were those that increase the UV-sensitivity of *rad7Δ* mutants. To assess whether this phenotype was general rather than specific to GG-NER, we generated double mutants of *rna14-1* with mutations in representative genes with known functions in the different DNA repair pathways, including homologous recombination (HR), non-homologous end joining (NHEJ), post-replicative repair (PRR), mismatch repair (MMR), base excision repair (BER) and NER (Figure 3B). Interestingly, the *rna14-1* mutant showed synthetic growth defects even in the absence of exogenous damage with several repair mutants, including *rad52Δ*, *ku70Δ*, *lig4Δ*, and *rad1Δ*. These growth defects are further sustained by DNA content profiling FACS analysis (Figure S5). In addition, synthetic UV/4-NQO sensitivity was observed in all double mutants but *rna14-1 ogg1Δ ntg1Δ ntg2Δ*. Thus, our results indicate that Rna14 dysfunction makes cells unable to cope with high levels of DNA damage and rely on functional repair pathways even in the absence of exogenous damage.

To check whether these genetic interactions might arise from expression defects of DNA repair genes, mRNA expression was analysed by microarrays in *rna14-1* and *rna15-1* cells (Table S1). The results obtained with the two mutants were highly similar (Figure S6). Analysis of gene ontology terms of genes with higher (> 2-fold) and lower (< 2-fold) expression as compared to wild-type levels revealed that many genes involved in the DNA damage and/or stress response are induced in these mutants (Table S2), including genes such as *OGG2*, *PRX1*, *DNL4*, *LIF1*, *RAD2* or *MAG1*. In addition, we found out that in *rna14-1* or *rna15-1* cells, the down-regulated genes were on the average longer than those of the entire genome, while the up-regulated genes were shorter (Figure S6), but DNA repair genes were not specifically down regulated. Thus the results rule out that the reduced capability of CFI mutants to withstand DNA damage is due to reduced transcription of repair protein encoding genes. On the contrary, the elevated expression of DNA damage and/or stress response transcripts suggests that CFI mutants may accumulate DNA damage or structures that impose a steric hindrance to DNA metabolic processes.

### CFI mutants show severe replication defects

**Figure 4:**
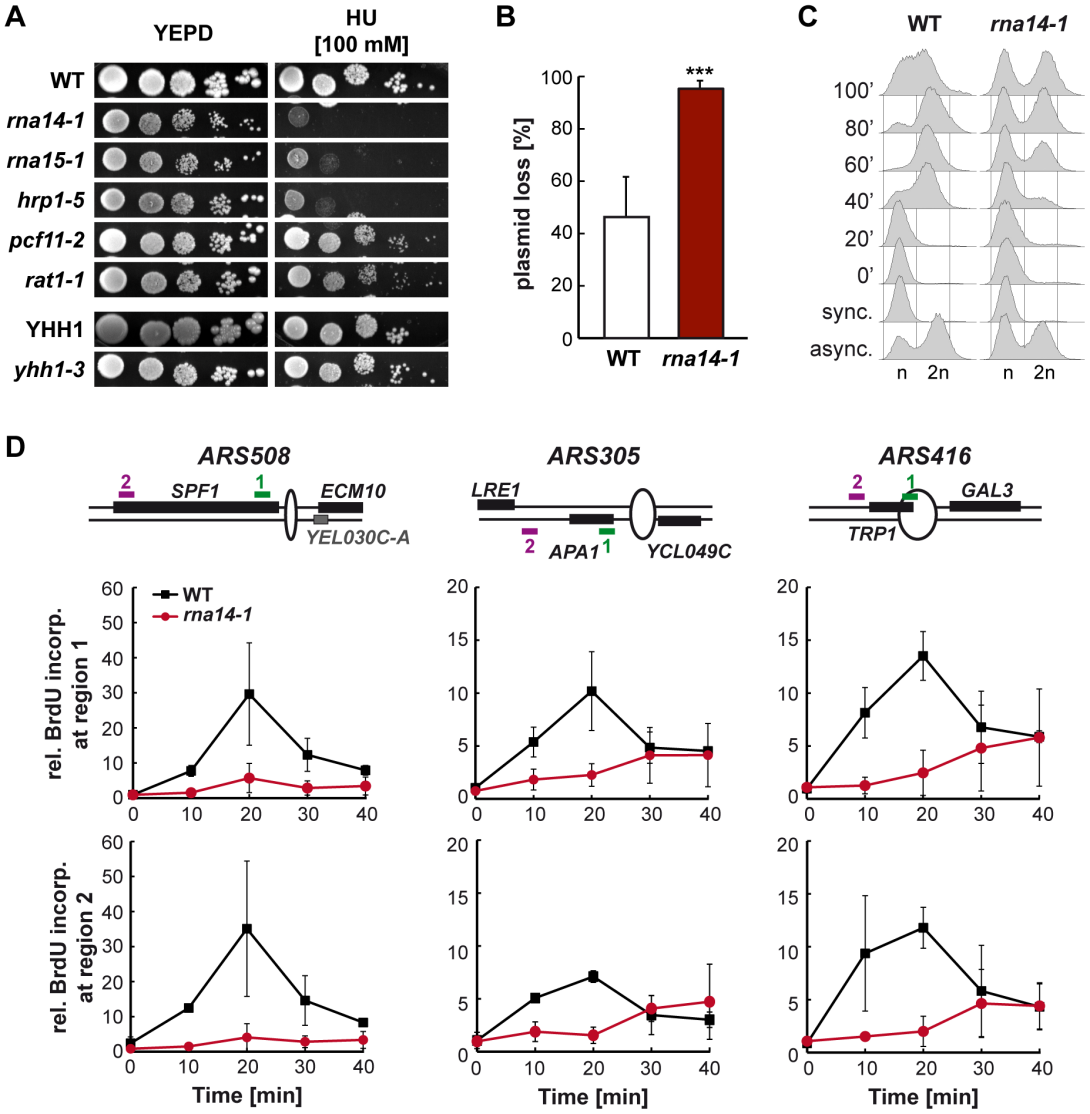
Cell cycle progression is compromised in *rna14-1* cells. (A) Sensitivity to hydroxyurea (HU) of six transcription-termination mutants. Serial dilutions (10-fold) of exponentially growing cultures are shown. (B) Analysis of plasmid loss in *rna14-1*, monitored as the percentage of cells that lost the pRS315 centromeric plasmid after ∼10 divisions in non-selective media. Average and standard deviation of six independent transformants are plotted for each genotype. Statistical analysis was performed with a two-tailed unpaired student t-test compared with the wild type. ***p<0.001. (C) Cell cycle progression analysis in wild-type (WT) and *rna14-1* strains. Asynchronous (async.), α-factor synchronized (sync.) and released cells were analysed by FACS. (D) Analysis of replication in *rna14-1* cells. BrdU incorporation upon release of G1-arrested cells was analysed at early replicating origins *ARS508*, *ARS305*, and *ARS416* by immunoprecipitation and RT qPCR. A schematic drawing of each *ARS* and localization of the amplified regions are depicted (top). Quantification of BrdU incorporation relative to a late replicating locus is plotted for each region. Average from two independent experiments and corresponding standard deviations are shown.

Transcription and replication need to occur in a coordinated manner in order to avoid conflicts that can result in genetic instability (reviewed in [30], [31]). To assess whether the CFI dysfunction affects replication, we first analysed sensitivity of several mutants to hydroxyurea (HU), a drug that slows replication down by reducing the pool of available deoxyribonucleotides (Figure 4A). Notably, the alleles that conferred sensitivity to HU were *rna14-1*, *rna15-1*, and *hrp1-5*, while the others did not at concentrations assayed. Since the expression of genes encoding ribonucleotide reductase components were not affected in *rna14-1* and *rna15-1* (Table S1), the observed HU sensitivity might reflect DNA replication impairment. Next we analysed plasmid loss in *rna14-1* cells as a way to measure replication efficiency genetically (Figure 4B). Our results show that less than 5% *rna14-1* cells maintained the pRS315 centromeric plasmid after about 10 divisions in non-selective medium as compared to the 50% value of wild-type cells. FACS analysis of cell cycle progression upon release from α-factor-mediated G1-arrest revealed that *rna14-1* mutants remain trapped in G1 and suffer from a delay in S-phase entry as compared to the wild type (Figure 4C). For a specific analysis of initiation and progression of replication, we monitored BrdU incorporation upon release from α-factor-mediated G1-arrest at three different early origins (Figure 4D). DNA was immunoprecipitated with anti-BrdU antibody and BrdU enrichment at each locus was analysed by real-time qPCR with specific primers. Importantly, strong defects in replication were observed in *rna14-1* mutants, as ARS activation was significantly reduced and occurred at later time points than in wild-type cells. Thus, cell-cycle progression is severely compromised in *rna14-1* cells.

### *rna14-1* relies on a functional G1/S checkpoint to avoid genomic instability

**Figure 5:**
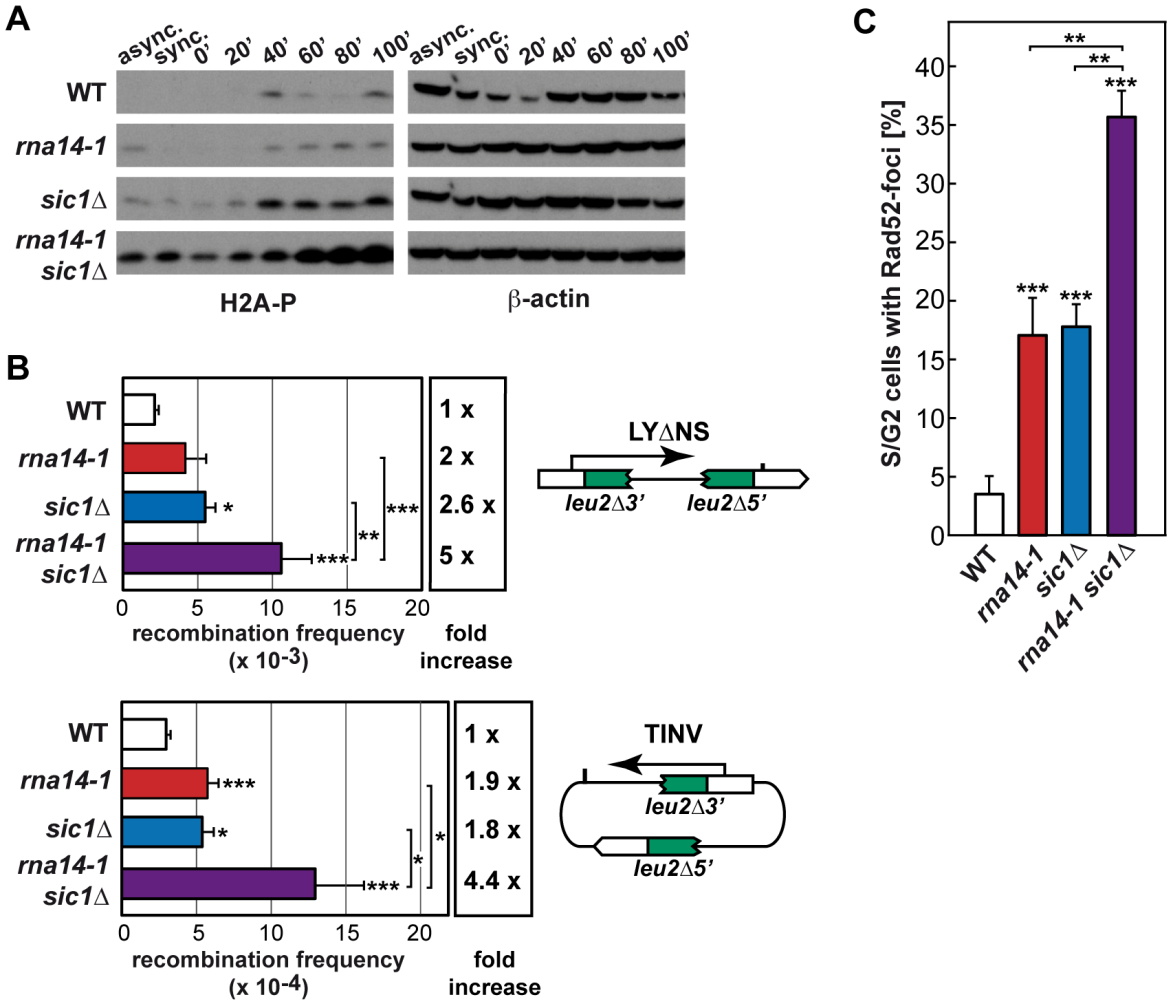
Absence of functional G1/S checkpoint leads to DNA damage and genomic instability in *rna14-1* cells. (A) Analysis of phosphorylated histone H2A (H2A-P) accumulation during release from α-factor-mediated G1-arrest in wild-type (WT), *rna14-1*, *sic1Δ* and *rna14-1 sic1Δ* strains. Asynchronous (async.), α-factor synchronized (sync.) and released cells were analysed. β-actin is shown as loading control. FACS analysis of all samples is shown in Figure S7. (B) Recombination analysis using a direct-repeat (LY*Δ*NS) and an inverted-repeat (TINV) plasmid-borne system. A scheme of each system is shown on the right of the corresponding panel. Recombination frequencies were obtained as the median value of six independent colonies. The average and standard deviation of at least three independent fluctuation tests are shown for each genotype. Statistical analyses were performed with a two-tailed unpaired student t-test compared with the wild type. Where indicated, statistical analyses between two mutants were also performed. *p<0.01, **p<0.005, ***p<0.001. (C) Percentage of S/G2 cells containing Rad52-YFP foci. Average of numbers obtained from at least three independent transformants and the corresponding standard deviation are shown. Statistical analyses as in B.

Because G1 to S-phase progression was markedly delayed in *rna14-1* cells, we asked whether persistent G1/S checkpoint activation might be responsible for the observed cell-cycle delay. Deprivation of Sic1, a protein that is required for the G1/S checkpoint, suppressed the S-phase entry defects in the *rna14-1* mutant upon release from α-factor-mediated G1-arrest as seen by FACS analysis (Figure S7). To evaluate the consequences of forcing S-phase entry in *rna14-1* mutants by *SIC1* deletion, we analysed phosphorylated H2A (H2A-P) levels by Western analysis (Figure 5A). Our results indicate that the *rna14-1 sic1Δ* mutant accumulates DNA damage, as seen by the large amount of H2A-P. We then analysed recombination and Rad52-foci accumulation to gain insight into the impact of G1/S-checkpoint bypass in *rna14-1* cells. As *rna14-1 sic1Δ* shows severe growth defects at 30°C (Figure S8), recombination was scored at 26°C, a semi-permissive temperature for the *rna14-1* mutant, in a direct-repeat (LY*Δ*NS) as well as an inverted-repeat (TINV) plasmid-based system [28] (Figure 5B). A significant increase in recombination frequency was observed in the double *rna14-1 sic1Δ* mutants with respect to the frequencies of either single mutant in both systems. Rad52-foci accumulation was monitored in cells transformed with plasmid pWJ1344 expressing a Rad52-YFP fusion protein using fluorescence microscopy. As can be seen in Figure 5C, the percentage of S/G2 cells with Rad52-foci was significantly higher in the *rna14-1 sic1Δ* double mutant (≈35%) than in the single mutants (<20%). Altogether, these results indicate that a functional G1/S checkpoint is essential to avoid genomic instability and/or cell death in *rna14-1* cells.

## Discussion

In this study, we asked whether transcription termination might contribute to DNA repair by TC-NER in *S. cerevisiae*. We found that the *rna14-1*, *rna15-1*, and *hrp1-5* alleles of CFI confer increased UV and 4-NQO sensitivities in the absence of GG-NER, but surprisingly do not affect CPD repair in a transcribed gene. Importantly, we show that both checkpoint activation and RNAPII degradation are delayed in UV-irradiated CFI-deficient cells and that the *rna14-1* mutation interacts genetically with mutations affecting several DNA repair pathway, including HR, NHEJ, MMR, PPR, and NER, in some cases even in the absence of exogenous DNA damage. Our data indicate that CFI participates in DDR in yeast and that this function is needed to cope with high amount of DNA damage. Additionally, we demonstrate that the *rna14-1* mutation leads to severe cell cycle progression hindrance and that a functional G1/S checkpoint becomes essential in restraining genomic instability when CFI function is impaired.

Although the precise mechanisms underlying termination downstream of poly(A) sites and 3′-end processing of RNAPII-transcribed genes remains unresolved, it certainly requires cooperation among several factors, including CFI, CPF, Pap1, Rat1 and even the RNAPII holoenzyme (reviewed in [32], [33]). CFIA is progressively recruited to RNAPII during elongation and peaks at poly(A) sites [34], [35]. Its role in transcription termination and 3′-end processing is recapitulated by ongoing transcription past poly(A) sites and *in vitro* cleavage and polyadenylation defects in CFI mutants [36]–[38]. The CFIB factor Hrp1 binds throughout transcribed genes [39] and displays *in vitro* cleavage and polyadenylation defects when mutated [40], [41]. We found that CFIA *rna14-1* and *rna15-1* as well as the CFIB *hrp1-5* alleles increased the UV and 4-NQO sensitivities of cells deficient in GG-NER and led to Phlemomycin and MMS sensitivities while the CFIA *pcf11-2*, CPF *yhh1-3*, and the *rat1-1* alleles did not (see Figures 1 and 3A). On the other hand, UV-induced Rpb1 degradation is impaired in *rna14-1*, *rna15-1* and *pcf11-2* but not in *hrp1-5* while Rad53-phosphorylation upon UV irradiation is delayed in *rna14-1*, *rna15-1* and *hrp1-5* but not in *pcf11-2* cells (Figures 2C, 2D and S3). Thus it appears that the penetrance of each particular mutation depends on the assayed phenotype. Indeed, different *pcf11* alleles differ in phenotype strength as seen by RNAPII chromatin immunoprecipitation (ChIP) on the *ADH1* and *PMA1* genes [42]. However, transcriptional read-through or 3′-end processing defects alone might not be sufficient to impair the DDR as ongoing transcription past poly(A) sites are also observed in *yhh1-3* and *rat1-1* mutants, and *yhh1-3* is deficient in 3′-end cleavage and polyadenylation as well [22], [36], [43]. One possibility could be that the requirement of CFI function for the DDR could rely on intrinsic sensing activity or specific interaction with DDR factors, thus enabling CFI to coordinate transcription termination and DDR.

UV irradiation was shown to lead to 3′-end processing inhibition along with targeted RNAPII degradation in human cells, these responses seemingly being mediated by direct interaction between CstF, the functional homologue of yeast CFI, and BRCA1/BARD1 [44], [45]. The link between DDR and 3′-end processing is further supported by the observations that partial depletion of the CstF-50 subunit leads to increased UV sensitivity, reduced ability to ubiquitinate RNAPII in response to UV and defects in CPD repair in human cells [46]. Our results show a notable divergence with respect to the human system though, as no CPD repair defects were observed in yeast CFI mutants (see Figure 2A and 2B). Another difference between yeast and human is the observation that poly-adenylated mRNAs get stabilized upon UV irradiation in yeast [47], while transcript deadenylation takes place under damaging conditions in humans, mediated by DNA damage-dependent physical interaction between CstF and the PARN deadenylase [48]. In addition, it has recently been shown that targeted variation of poly(A) site usage occurs in response to 4-NQO treatment in yeast, possibly as a consequence of transient depletion of CPF subunits [49]. Altogether, these findings suggest that transcription termination factors participate in DDR, a multiple-sided system fundamental for cell survival under genotoxic stress conditions.

The cellular response to UV damage involves global down-regulation of transcriptional activity concomitantly with high expression of a subset of stress-induced genes and proteosomal-mediated degradation of RNAPII major subunit Rpb1. Notably, UV-induced Rpb1 degradation is delayed in CFI-deficient cells (see Figures 2C, 2D and S3), RNAPII turnover being thus impaired. Interestingly, transcription termination factors - including CFI - interact with the transcription initiation factor TFIIB and this interaction is required for the formation of gene loops both in yeast and humans [50]–[53]. Gene looping has been proposed to enable the efficient recycling of RNAPII and to contribute to transcription regulation by acting on promoter directionality and transcriptional memory (reviewed in [54], [55]). It is thus conceivable that gene looping may also function to control transcription and RNAPII turnover under DNA damaging conditions. This idea is supported by recent work showing that TFIIB may function as a general transcriptional switch in humans, as it is dephosphorylated during genotoxic stress thus losing its interaction with CstF, while direct interaction between CstF and the p53 tumor suppressor ensures the recruitment of termination factors to the promoter of stress-induced genes [56].

The persistence of stalled RNAPII on transcribed genes is known to impede the progression of the replication machinery and to be one of the causes underlying transcription-associated recombination (TAR) (reviewed in [30], [31]). Recently, inhibition of Rho-dependent transcription termination in bacteria has been shown to induce double-strand breaks depending on replication, suggesting that Rho might function in the release of obstructing RNAP during replication [57]. It is possible that CFI might act on paused RNAP, whether or not stalled at a DNA damage, contributing to its displacement and thus allowing progression of an oncoming replication fork. Over the last few years, growing evidence supports a role for co-transcriptionally formed RNA-DNA hybrids (R-loops) as a source of TAR (reviewed in [58]). Noteworthy, several transcription termination and 3′-end processing mutants have been shown to accumulate R-loops in yeast (including *pcf11-2* and *rna15-58*) [59]. It is thus possible that stalled RNAPIIs accumulate at DNA damages or other structures such as R-loop in CFI mutants, leading to steric hindrances to the replication machinery that would account for the observed cell cycle progression defects (see Figure 4). The mechanisms by which stalled RNAPIIs or structures presenting steric hindrance to replication are sensed to activate the G1/S cell cycle checkpoint, which is required to restrain genetic instability in *rna14-1* cells (see Figure 5), are currently unknown. Interestingly, the Sen1/SETX helicase - a component of the NRD transcription termination complex - prevents R-loop accumulation at transcription termination sites both in yeast and humans [60], [61]. In addition to its association with transcribed units, yeast Sen1 is also found at replication forks, contributing to prevent deleterious outcomes of the putative collisions between the transcription and replication machineries [62]. Noteworthy, Sen1 interacts physically with the NER repair protein Rad2 and the *sen1-1* mutation increases the UV sensitivity of cells lacking *RAD2* [63], suggesting further connections between transcription termination, replication, and DNA repair.

**Figure 6:**
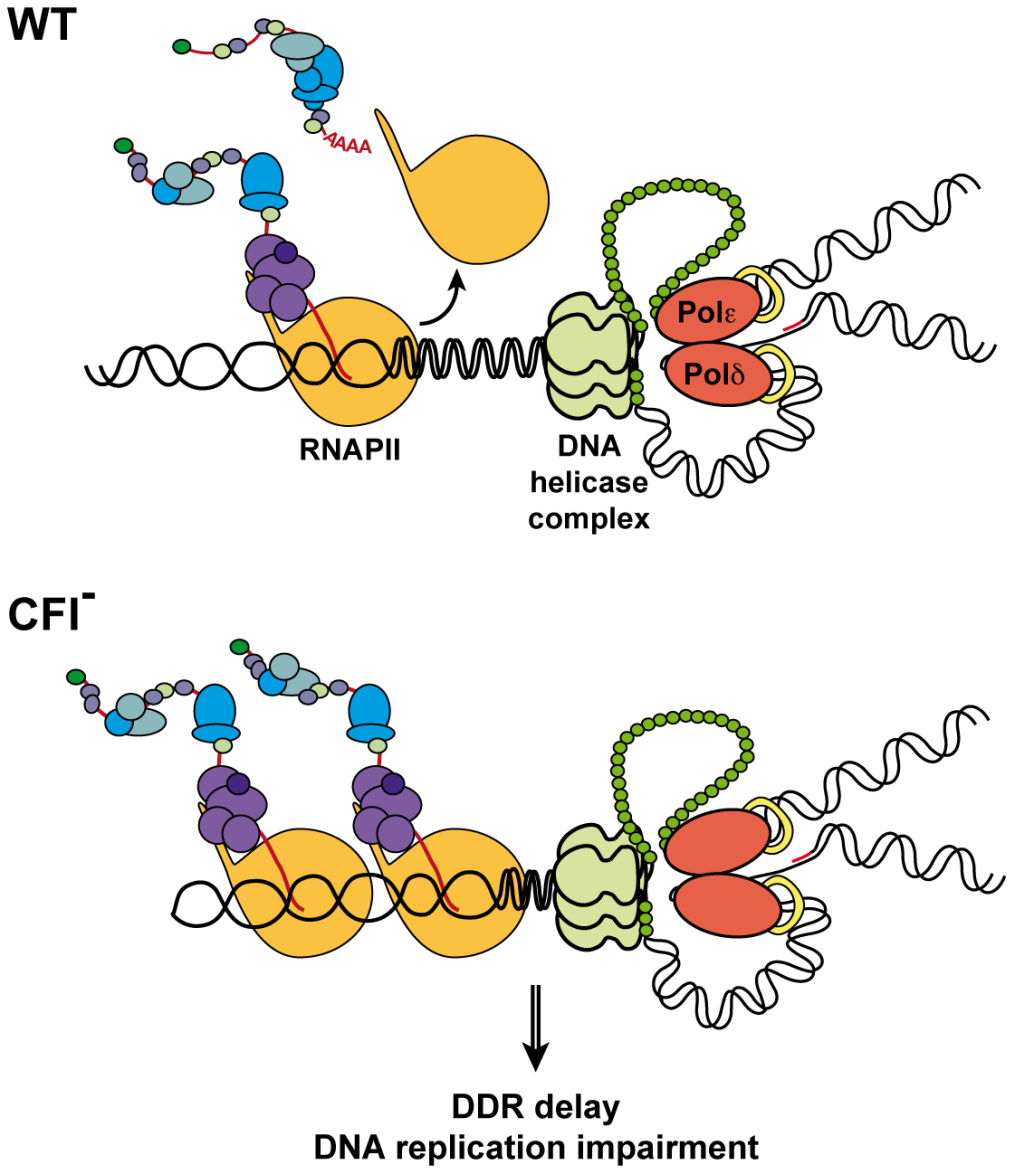
Model for concurrent transcription termination and replication processes. In wild-type cells (WT), transcription and replication are coordinated to prevent collision between both machineries and genomic instability. CFI function allows prompt DDR in the presence of DNA lesions. In CFI mutants (CFI^-^), impaired transcription termination interferes with replication and DDR is delayed. As a consequence, functional DNA repair pathways and G1/S checkpoint become crucial in those cells.

Altogether, our results support a model in which CFI dysfunction impairs DDR, probably leading to the accumulation of endogenous DNA lesions, and hinders DNA replication possibly due to the accumulation of RNAPs, whether or not stalled at DNA damages, thus rendering the G1/S checkpoint mandatory to prevent genomic instability (see Figure 6). Our findings emphasize the importance of coordinating transcription termination, DDR and replication in the maintenance of genomic stability and suggest that CFI plays a fundamental function in the coupling of these processes.

## Materials and Methods

### Yeast strains and plasmids

All strains used were isogenic to W303, and are listed in Table S3. Newly generated strains were obtained either by direct transformation or by genetic crosses. Plasmids used for recombination tests were pRS314-LY*Δ*NS, pRS316-TINV, pRS314-LY and pRS314-SU [28].

### UV survival curves and assays

For cell survival, yeast cells were grown in YEPD rich medium to an OD_600_ of 0.6. 10-fold serial dilutions were dropped on YEPD plates, irradiated with the indicated dose of UV-C light, and incubated in the dark at 30°C for 3 days. For the 4-NQO, Phleomycin, MMS, CPT and HU sensitivity assays, the serial dilutions were dropped on YEPD plates containing the indicated amounts of genotoxic agents and incubated in the dark at 30°C for 3 days. UV survival curves were performed as described [9]. UV-C irradiation was performed using a BS03 UV irradiation chamber and UV-Mat dosimeter (Dr. Gröbel UV-Elektronik GmbH).

### Gene- and strand-specific repair assays

CPD repair at the *RPB2* gene was analysed as described [64]. Briefly, cells were grown at 30°C in YEPD rich medium, irradiated in SD medium w/o amino acids with 200 J/m^2^ UV-C light (BS03 UV irradiation chamber), the medium supplemented to YEPD rich and the cells incubated at 30°C in the dark for recovery. DNA from the different time-points was extracted, cut with *Nsi*I and *Pvu*II restriction enzymes (Roche) and aliquots were either treated with T4-endonuclease V (Epicentre) or left untreated. DNA was electrophoresed in 1.3% alkaline agarose gels, blotted to Nylon membranes and hybridized with radioactively labelled strand-specific DNA probes, which were obtained by primer extension. Sequences of the primers are listed in Table S4. Membranes were analysed and quantified with a PhosphorImager (Fujifilm FLA5100). The average of the initial damage generated was 0.025 CPD/kb. To allow direct comparison between different strains, repair curves were calculated as the fraction of CPDs removed versus time. The initial damage was set to 0% repair.

### Expression microarray analysis

Cells were grown at 30°C in YEPD medium to an OD_660_ of 0.6. Total RNAs were purified (RNeasy Midi kit, Qiagen) and expression profiling performed using the Affymetrix platform (see Table S1). The relative RNA levels for all yeast genes were determined using an Affymetrix microarray scanner and processed using the robust multiarray average method. Statistical data analyses were performed using the limma package (affylmGUI interface) of the R Bioconductor project (http://www.bioconductor.org). For each strain, microarray analysis was conducted in triplicate. All values presented represent the average of these three determinations. Genes were considered significantly up- or down-regulated when their expression values were > or < 2-fold, respectively (parameters: false discovery rate-adjusted p-value<0.01, B-statistic value>2, and average log2intensity A>7). The expression data for each mutant has been deposited in NCBI's Gene Expression Omnibus (accession number GSE50947).

### Recombination and plasmid-loss assays

Plasmid loss was monitored as the percentage of cells that lost centromeric plasmid pRS315 upon growth in non-selective media. Individual transformants were inoculated in 5 ml YEPD and grown at 30°C to OD_660_ 0.6. Cells were plated on YEPD or SC-leu to determine the percentage of plasmid loss. Six individual transformants were analysed for each genotype.

Recombination frequencies were determined as the average value of the median frequencies obtained from at least three independent fluctuation tests performed at 26°C each from six independent colonies according to standard procedures [28].

### Replication analysis

Isogenic wild-type and *rna14-1* strains deleted for the *BAR1* gene and carrying several copies of the Herpes simplex thymidine kinase (TK) under the control of the strong constitutive *GPD* promoter were obtained by genetic crosses with strain SY2201 (E. Schwob). Cells were grown in YEPD, incubated for 2.5 h with 0.125 µg/ml α-factor, washed twice with pre-warmed YEPD and released into S phase by addition of 1 µg/ml pronase. BrdU (200 µg/ml) was added to the cultures prior to release. Cell cycle progression was monitored by flow cytometry on a FACSCalibur (BD Bioscience) using CellQuest software. Chromatin immunoprecipitation was carried out as described [65] with minor modifications. Briefly, Sodium Azide (0.1%) was added to each sample and cells were broken in a multi-beads Shocker (MB400U, Yasui Kikai, Japan) at 4° in lysis buffer (50 mM HEPES-KOH pH 7.5, 140 mM NaCl, 1 mM EDTA, 1% triton X-100, 0.1% sodium deoxicholate) and sonicated. Immunoprecipitation was performed using anti-BrdU antibody (MBL) attached to magnetic beads coated with Protein A (Invitrogen). Input and precipitated DNA were analysed by RT qPCR (7500FAST Applied Biosystems). Relative BrdU incorporation at a given region was calculated relative to the signal at a late replicating region (Chr. V, position 242210–242280, [66]) in the same sample. Primer sequences are listed in Table S4.

### Detection of Rad52-YFP

Rad52-YFP foci from log-phase cells transformed with plasmid pWJ1344 were visualized with a DM600B microscope (Leica) as previously described [67] with minor modifications. Individual transformants were grown to early-log-phase at 26°C, incubated at 30°C for 4 hours, fixed for 10 minutes in 0.1 M K_i_PO_4_ pH 6.4 containing 2.5% formaldehyde, washed twice in 0.1 M K_i_PO_4_ pH 6.6, and resuspended in 0.1 M K_i_PO_4_ pH 7.4. A total of 617 wild type, 947 *rna14-1*, 733 *sic1Δ*, and 820 *rna14-1 sic1Δ* cells derived from at least three different transformants were analysed.

### Cell extracts and western analysis

Detection of Rpb1, Rad53, H2A-P, and β-actin was accomplished by Western analysis of TCA-precipitated proteins separated in 4–20% Cristerion TGX gradient PAGE (Biorad). Antibodies 8WG16 (Rpb1, Covance), sc-20169 (Rad53, Santa Cruz Biotechnology), ab15083 (H2A-P, Abcam) and ab8224 (β-actin, Abcam) were used. For quantification, secondary antibodies conjugated to IRDye 680CW or 800CW (LI-COR) were used, the blot scanned in an Odyssey IR scanner and analysed with Image Studio 2.0 software (LI-COR). For Western analysis after UV irradiation, cells were grown in YEPD rich medium to mid-log-phase, resuspended in SD media lacking amino acids to an OD_660_ of 0.6 and irradiated with UV-C light in a BS03 UV irradiation chamber (Dr. Gröbel UV-Elektronik GmbH) at 100 J/m^2^. Medium was supplemented to YEPD rich and cells incubated in the dark at 30°C for recovery.

## Supporting Information

Figure S1 Recombination rates of *rna14-1* cells do not increase upon UV irradiation. Recombination analysis using a direct-repeat (LY) and an inverted-repeat (SU) plasmid-borne systems in wild-type (WT), *rad7Δ*, *rna14-1* and *rna14-1 rad7Δ* strains with or without UV irradiation. A scheme of each system is shown on top of the corresponding panel. Recombination frequencies were obtained as the median value of six independent colonies. The average and standard deviation of at least three independent fluctuation tests are shown for each condition. Statistical analyses were performed with a two-tailed unpaired student t-test compared with the wild type. *p<0.01, **p<0.005, ***p<0.001.
(TIF)

Figure S2 *rna14-1* and DNA damage checkpoint mutants do not show genetic interactions. Analysis of genetic interactions between *rna14-1* and mutants impaired in DNA damage checkpoint and sensitivity to UV and 4-NQO. 10-fold serial dilutions of exponentially growing cultures are shown.
(TIF)

Figure S3 DNA-damage response alteration in transcription termination mutants. (A) Western analysis of Rpb1 and Rad53 upon UV irradiation in *rna15-1*, *hrp1-5* and *pcf11-2* cells. β-actin is shown as loading control. (B) Graphical representation of the quantified results from Rpb1 and Rad53 Western analyses. The amount of Rpb1 is shown as the percentage of Rpb1 in the non-irradiated sample. The percentage of hyper-phosphorylated Rad53 is plotted for each condition. Average values derived from two independent experiments are plotted with their standard deviation.
(TIF)

Figure S4 Transcription termination mutants show synthetic growth defects with *def1Δ*. Analysis of genetic interactions between four transcription termination deficient alleles and the *def1Δ* mutation. 10-fold serial dilutions of exponentially growing cultures are shown. Note that the data of wild-type, *def1Δ*, *rna14-1* and *rna14-1 def1Δ* strains is also shown in Figure 2E.
(TIF)

Figure S5 Analysis of genetic interactions between *rna14-1* and DNA repair mutants. DNA contents profile of *rna14-1* and mutants impaired in homologous recombination (*rad52Δ*), non-homologous end joining (*ku70Δ* and *lig4Δ*), post-replicative repair (*rad18Δ*), mismatch repair (*msh2Δ*), base excision repair (*ogg1Δ ntg1Δ ntg2Δ*), and nucleotide excision repair (*rad1Δ*) analysed by FACS.
(TIF)

Figure S6 Comparative analysis of up- and down-regulated genes in *rna14-1* and *rna15-1* cells. (A) Venn diagrams representing the overlap between genes whose expression is changed more than 2-fold with respect to the wild type in *rna14-1* and *rna15-1* mutants. (B) Linear regression and corresponding equation is shown for the *rna14-1* and *rna15-1* data sets. (C) Statistical analysis of length of genes whose expression level changes in *rna14-1* and *rna15-1* as compared with the genome average.
(TIF)

Figure S7 Absence of functional G1/S checkpoint forces *rna14-1* cells to enter S-phase. Cell cycle progression analysis in wild-type (WT), *rna14-1*, *sic1Δ* and *rna14-1 sic1Δ* strains upon release from α-factor-mediated G1-arrest. Asynchronous (async.), α-factor synchronized (sync.) and released cells were analysed by FACS. Positions of n and 2n peaks are indicated.
(TIF)

Figure S8 Temperature sensitivity of *rna14-1 sic1Δ* double mutants. Growth of wild-type (WT), *rna14-1*, *sic1Δ* and *rna14-1 sic1Δ* strains at 26°C and 30°C on YEPD plates.
(TIF)

Table S1 List of genes with altered expression levels in *rna14-1* and *rna15-1* mutants.
(XLSX)

Table S2 Gene ontology results for the genes with altered expression levels in *rna14-1* and *rna15-1* mutants.
(PDF)

Table S3 Yeast strains used in this study.
(PDF)

Table S4 Primers used in this study.
(PDF)
