# Supplementary figures and images for: Cleavage Factor I Links Transcription Termination to DNA Damage Response and Genome Integrity Maintenance in Saccharomyces cerevisiae
Source: PLoS Genet. 2014 Mar 6;10(3):e1004203. doi: 10.1371/journal.pgen.1004203 (PMC3945788; doi:10.1371/journal.pgen.1004203)

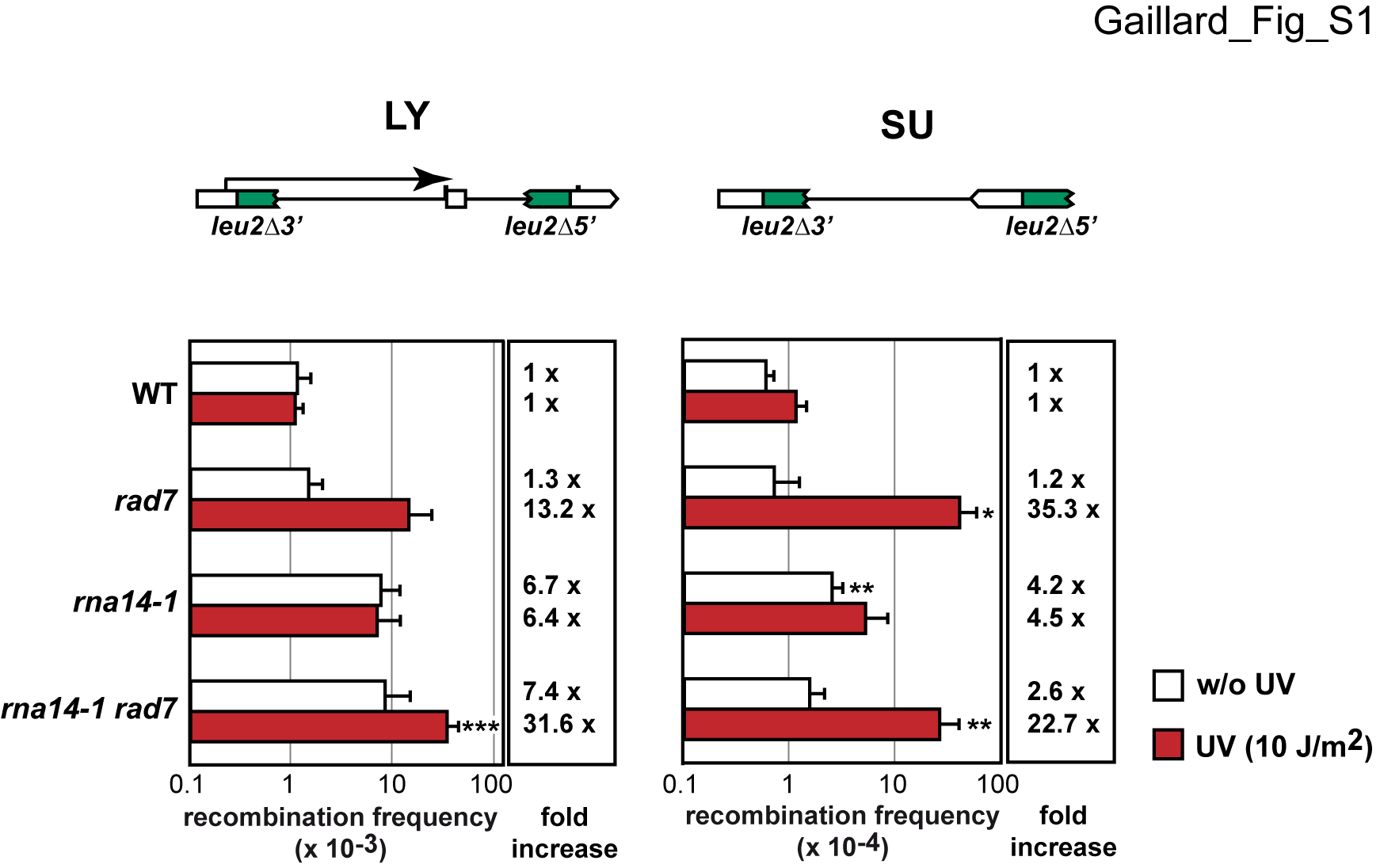

Supplement: Figure S1 — Recombination rates of rna14-1 cells do not increase upon UV irradiation. Recombination analysis using a direct-repeat (LY) and an inverted-repeat (SU) plasmid-borne systems in wild-type (WT), rad7Δ, rna14-1 and rna14-1 rad7Δ strains with or without UV irradiation. A scheme of each system is shown on top of the corresponding panel. Recombination frequencies were obtained as the median value of six independent colonies. The average and standard deviation of at least three independent fluctuation tests are shown for each condition. Statistical analyses were performed with a two-tailed unpaired student t-test compared with the wild type. *p<0.01, **p<0.005, ***p<0.001. (TIF) [file pgen.1004203.s001.tif]

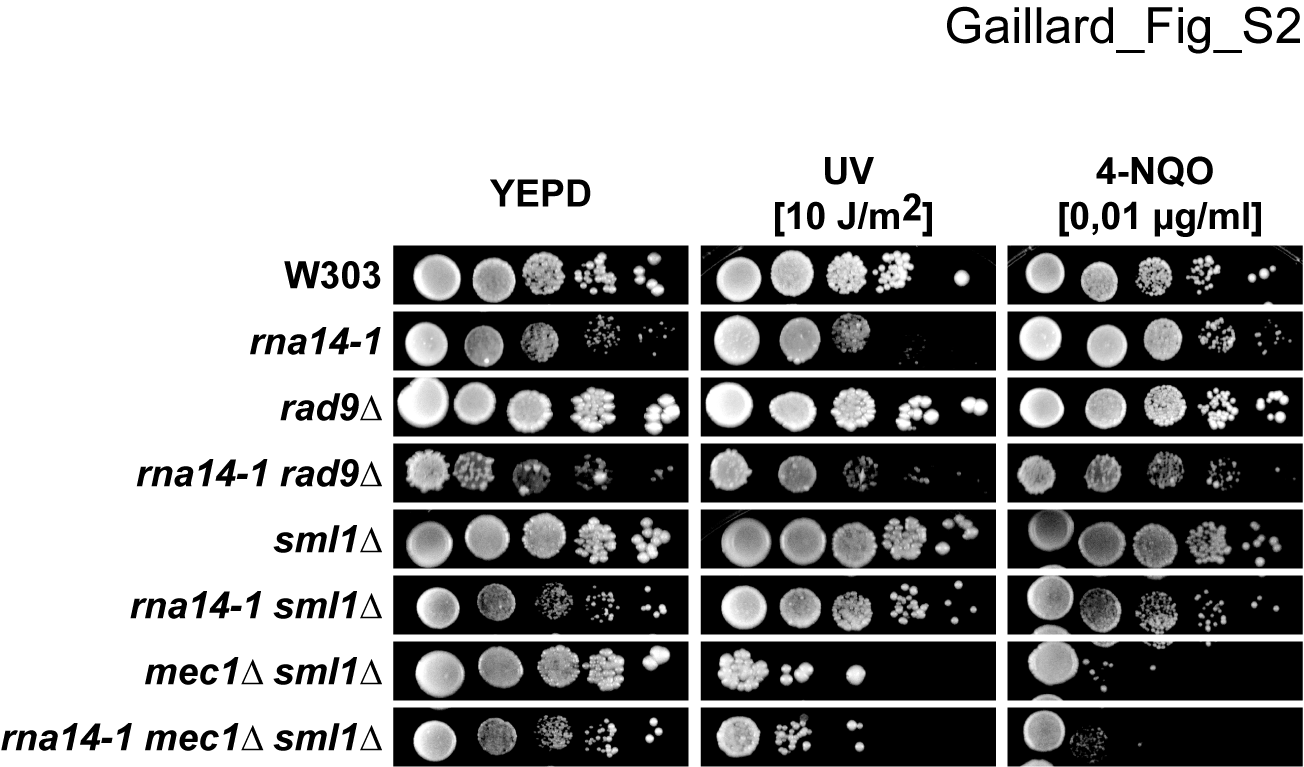

Supplement: Figure S2 — rna14-1 and DNA damage checkpoint mutants do not show genetic interactions. Analysis of genetic interactions between rna14-1 and mutants impaired in DNA damage checkpoint and sensitivity to UV and 4-NQO. 10-fold serial dilutions of exponentially growing cultures are shown. (TIF) [file pgen.1004203.s002.tif]

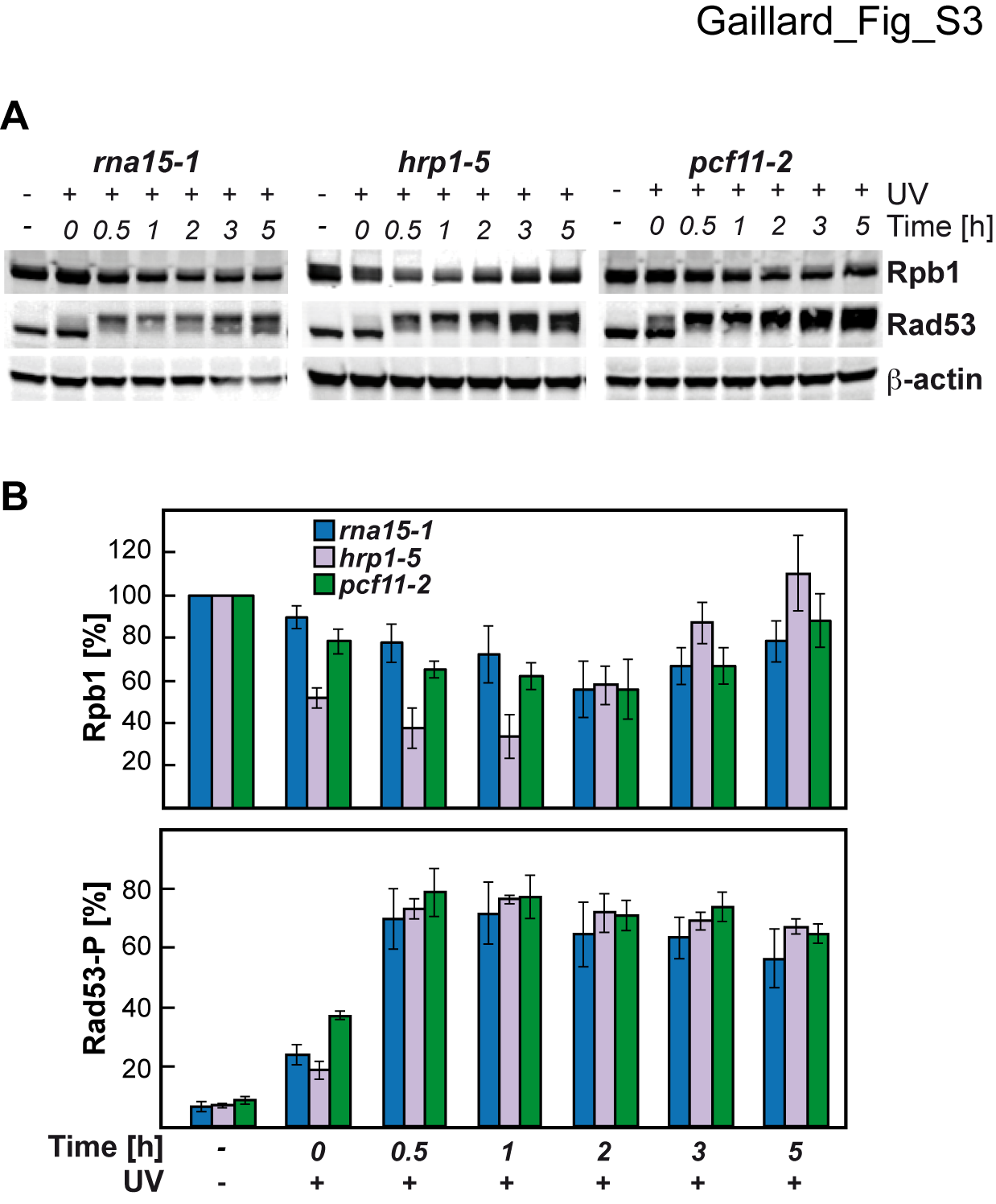

Supplement: Figure S3 — DNA-damage response alteration in transcription termination mutants. (A) Western analysis of Rpb1 and Rad53 upon UV irradiation in rna15-1, hrp1-5 and pcf11-2 cells. β-actin is shown as loading control. (B) Graphical representation of the quantified results from Rpb1 and Rad53 Western analyses. The amount of Rpb1 is shown as the percentage of Rpb1 in the non-irradiated sample. The percentage of hyper-phosphorylated Rad53 is plotted for each condition. Average values derived from two independent experiments are plotted with their standard deviation. (TIF) [file pgen.1004203.s003.tif]

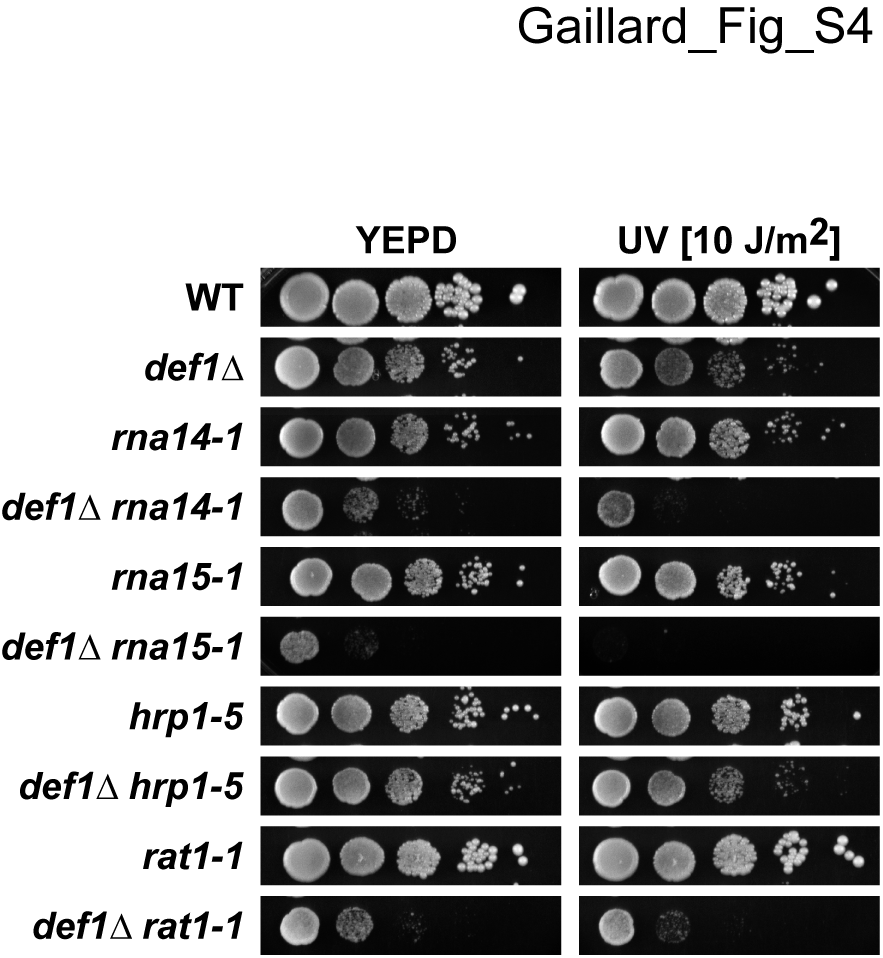

Supplement: Figure S4 — Transcription termination mutants show synthetic growth defects with def1Δ. Analysis of genetic interactions between four transcription termination deficient alleles and the def1Δ mutation. 10-fold serial dilutions of exponentially growing cultures are shown. Note that the data of wild-type, def1Δ, rna14-1 and rna14-1 def1Δ strains is also shown in Figure 2E. (TIF) [file pgen.1004203.s004.tif]

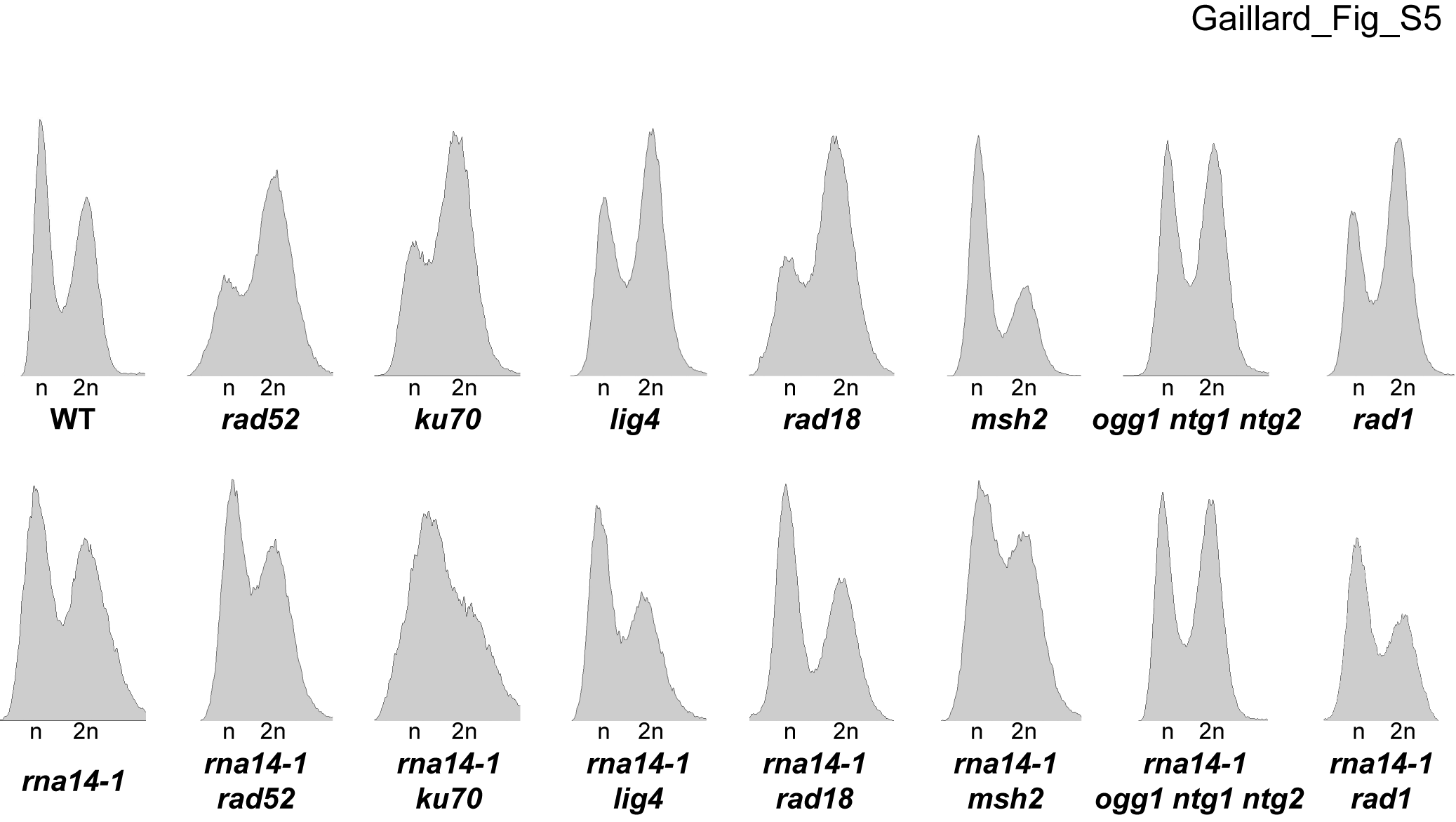

Supplement: Figure S5 — Analysis of genetic interactions between rna14-1 and DNA repair mutants. DNA contents profile of rna14-1 and mutants impaired in homologous recombination (rad52Δ), non-homologous end joining (ku70Δ and lig4Δ), post-replicative repair (rad18Δ), mismatch repair (msh2Δ), base excision repair (ogg1Δ ntg1Δ ntg2Δ), and nucleotide excision repair (rad1Δ) analysed by FACS. (TIF) [file pgen.1004203.s005.tif]

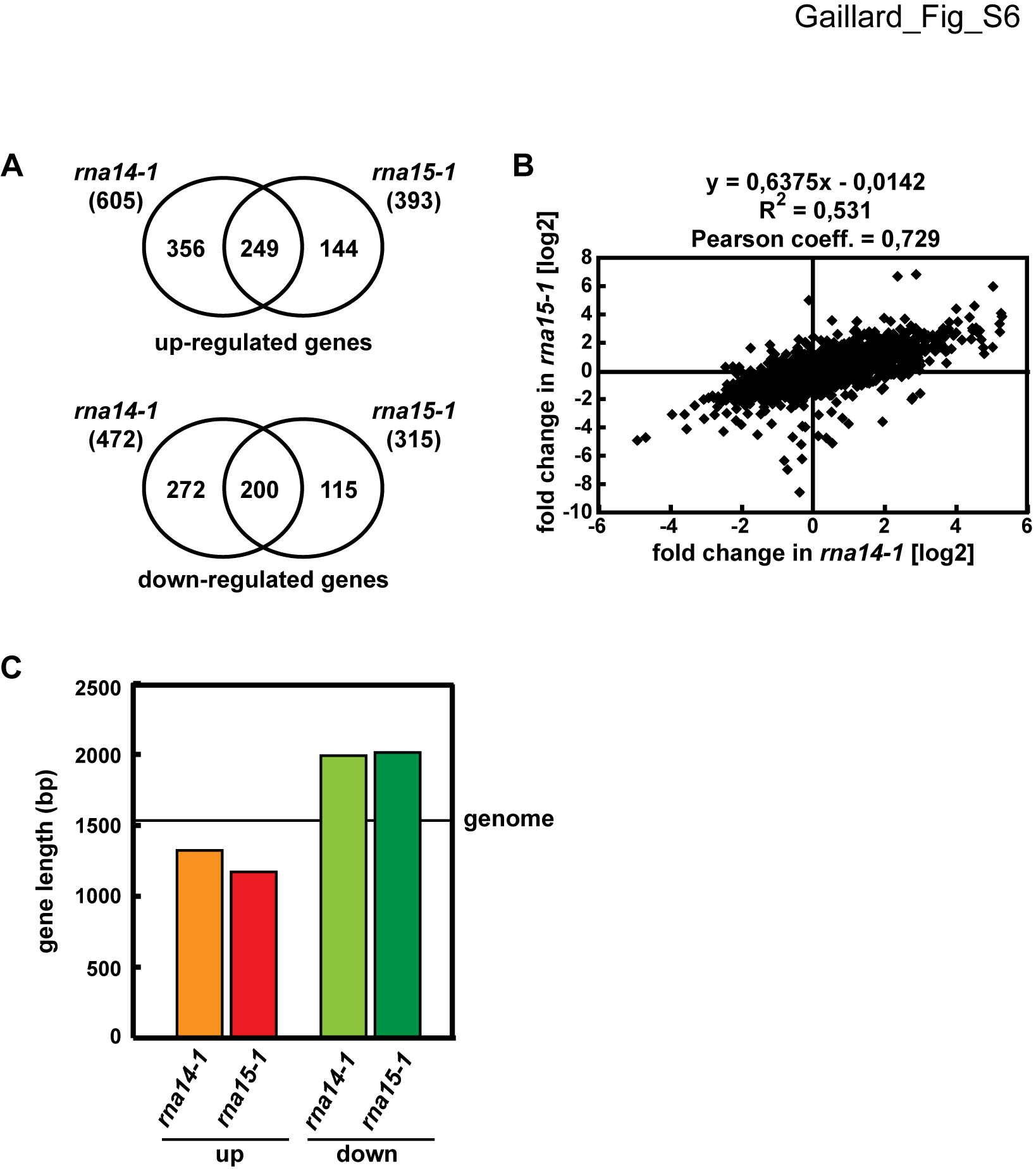

Supplement: Figure S6 — Comparative analysis of up- and down-regulated genes in rna14-1 and rna15-1 cells. (A) Venn diagrams representing the overlap between genes whose expression is changed more than 2-fold with respect to the wild type in rna14-1 and rna15-1 mutants. (B) Linear regression and corresponding equation is shown for the rna14-1 and rna15-1 data sets. (C) Statistical analysis of length of genes whose expression level changes in rna14-1 and rna15-1 as compared with the genome average. (TIF) [file pgen.1004203.s006.tif]

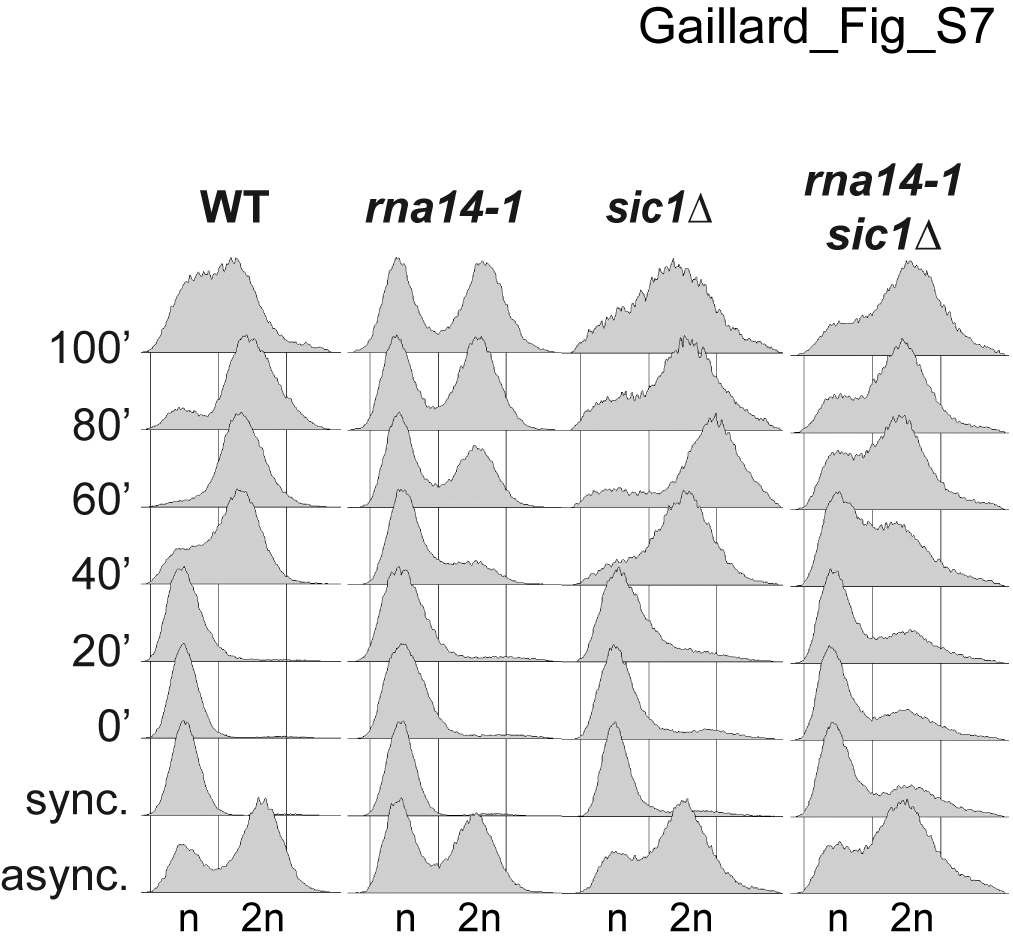

Supplement: Figure S7 — Absence of functional G1/S checkpoint forces rna14-1 cells to enter S-phase. Cell cycle progression analysis in wild-type (WT), rna14-1, sic1Δ and rna14-1 sic1Δ strains upon release from α-factor-mediated G1-arrest. Asynchronous (async.), α-factor synchronized (sync.) and released cells were analysed by FACS. Positions of n and 2n peaks are indicated. (TIF) [file pgen.1004203.s007.tif]

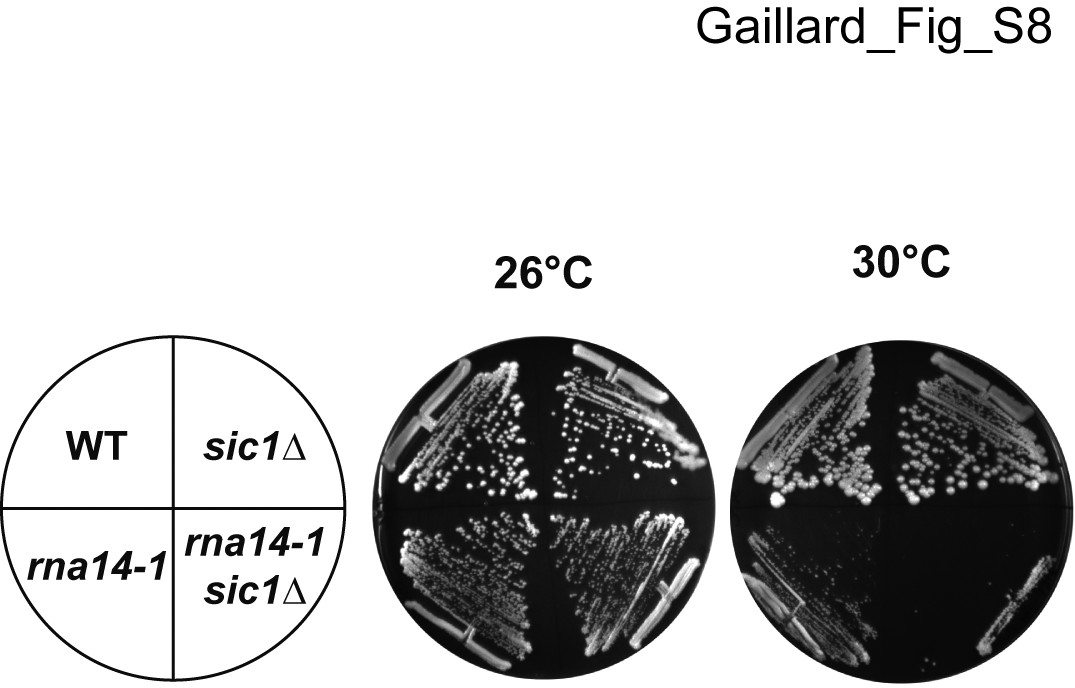

Supplement: Figure S8 — Temperature sensitivity of rna14-1 sic1Δ double mutants. Growth of wild-type (WT), rna14-1, sic1Δ and rna14-1 sic1Δ strains at 26°C and 30°C on YEPD plates. (TIF) [file pgen.1004203.s008.tif]
