# Supplementary material for: Cleavage Factor I Links Transcription Termination to DNA Damage Response and Genome Integrity Maintenance in Saccharomyces cerevisiae
Source: PLoS Genet. 2014 Mar 6;10(3):e1004203. doi: 10.1371/journal.pgen.1004203 (PMC3945788; doi:10.1371/journal.pgen.1004203)
Supplement: Table S2 — Gene ontology results for the genes with altered expression levels in rna14-1 and rna15-1 mutants. (PDF) [file pgen.1004203.s010.pdf]

Table S2. Gene Ontology results for the genes with altered expression levels in *rna14-1* and *rna15-1* mutants.

	GO term (GO ID) of up-regulated genes	*rna14-1* P-value	*rna15-1* P-value
**catabolic processes**	catabolic process (9056)	3.07e-06	
	cofactor catabolic process (51187)		0.00599
	carboxylic acid catabolic process (46395)	0.00394	
	mitochondrion degradation (422)	0.00753	
**response to stimulus**	response to stimulus (50896)	1.04e-05	0.00338
	response to stress (6950)	0.00137	
	response to chemical stimulus (42221)	0.00830	5.07e-06
	response to organic substance (10033)		0.00325
**reproductive process**	sexual reproduction (19953)	0.00329	4.79e-08
	sporulation (43934)	0.00066	
	reproductive process (22414/22413)	0.00690	1.25e-07
	conjugation with cellular fusion (747)		1.63e-06
	response to pheromone (19236)		3.96e-05
**Cell development**	cell differentiation (30154)	0.00389	
	anatomical structure formation involved in morphogenesis (48646)	0.00066	
	anatomical structure development (48856)	0.00231	
**others**	energy derivation by oxidation of organic compounds (15980)	0.00312	
	tricarboxylic acid cycle (6099)		0.00764
	glucose metabolic process (6006)	0.00519	
	multi-organism process (51704)		4.19e-06

	GO term (GO ID) of down-regulated genes	*rna14-1* P-value	*rna15-1* P-value
**localization**	establishment of localization (51234)	0.00825	7.61e-08
	localization (51179)	0.00182	1.40e-08
**glycoprotein processes**	glycoprotein biosynthetic process (9101)	1.26e-07	0.00224
	macromolecule glycosylation (43413)	1.84e-07	0.00093
	protein amino acid glycosylation (6486)	1.84e-07	0.00093
	glycosylation (70085)	1.84e-07	0.00093
	glycoprotein metabolic process (9100)	2.31e-07	0.00312
	protein amino acid O-linked glycosylation (6493)	0.00132	
	protein amino acid N-linked glycosylation (6487)	0.00785	
**lipoprotein processes**	lipoprotein metabolic process (42157)	0.00027	
	lipoprotein biosynthetic process (42158)	0.00027	
	protein amino acid lipidation (6497)	0.00027	
**transport**	transport (6810)		1.13e-07
	transition metal ion transport (41)		2.60e-06
	metal ion transport (30001)		6.79e-06
	copper ion import (15677)		0.00013
	copper ion transmembrane transport (35434)		0.00037
	cation transport (6812)		0.00082
	copper ion transport (6825)		0.00100
	ion transport (6811)		0.00543
	ion transmembrane transport (34220)		0.00590
	transmembrane transport (55085)		0.00841
	iron ion transport (6826)		0.00999
**others**	cellular process (9987)	0.00073	
	cellular carbohydrate metabolic process (44262)	0.00088	
	carbohydrate metabolic process (5975)	0.00237	
