# Supplementary material for: Cleavage Factor I Links Transcription Termination to DNA Damage Response and Genome Integrity Maintenance in Saccharomyces cerevisiae
Source: PLoS Genet. 2014 Mar 6;10(3):e1004203. doi: 10.1371/journal.pgen.1004203 (PMC3945788; doi:10.1371/journal.pgen.1004203)
Supplement: Table S3 — Yeast strains used in this study. (PDF) [file pgen.1004203.s011.pdf]

**Table S3. Yeast strains used in this study.**

| Strain | Relevant genotype | Reference |
|---|---|---|
| W303-1A | *MAT**a** ura3-1 ade2-1 leu2-3,112 trp1-1 his3-11,15* | R. Rothstein |
| W303-1B | *MATa ura3-1 ade2-1 leu2-3,112 trp1-1 his3-11,15* | R. Rothstein |
| *rna14-1* W | *MATa ura3-1 ade2-1 leu2-3,112 trp1-1 his3-11,15 rna14-1* | [1] |
| *rna15-1* W | *MATa ura3-1 ade2-1 leu2-3,112 trp1-1 his3-11,15 rna15-1* | F. Lacroute |
| NA65 | *MAT**a** ura3-1 trp1Δ ade2-1 leu2-3,112 his3-11,15 pcf11-2* | [1] |
| MHY300 | *MATa ura3-1 ade2-1 leu2-3,112 his3-11,15 trp hrp1-5* | C. Moore |
| YBD40 | *ura3 ade2-1 leu2,3-112 his3-11,15 trp1Δ yhh1Δ::TRP1* pBD57 (*YHH1-URA3-CEN*) | [2] |
| YBD57 | *ura3 ade2-1 leu2,3-112 his3-11,15 trp1Δ yhh1Δ::TRP1* (*yhh1-3-LEU2-CEN*) | [2] |
| YHG104-6B | *MAT**a** ura3-1 ade2-1 leu2-3,112 trp1-1 his3-11,15 rat1-1* | This study |
| MGSC97 | *MATa ura3-1 trp1-1 ade2-1 leu2-3,112 his3-11,15 rad7Δ::URA3* | [3] |
| yMR01 | *MATa ura3-1 ade2-1 leu2-3,112 trp1-1 his3-11,15 rna14-1 rad7Δ::URA3* | This study |
| yHG50 | *MATa ura3-1 ade2-1 leu2-3,112 trp1-1 his3-11,15 rna14-1 rad7Δ::KAN* | This study |
| yHG51 | *MATa ura3-1 ade2-1 leu2-3,112 trp1-1 his3-11,15 rna15-1 rad7Δ::KAN* | This study |
| yHG52 | *MATa ura3-1 ade2-1 leu2-3,112 his3-11,15 trp hrp1-5 rad7Δ::KAN* | This study |
| yMR02 | *MAT**a** ura3-1 trp1-1 ade2-1 leu2-3,112 his3-11,15 pcf11-2 rad7Δ::URA3* | This study |
| yHG53 | *ura3 ade2-1 leu2,3-112 his3-11,15 trp1Δ yhh1Δ::TRP1* pBd57 (YHH1 ura3 CEN) *rad7Δ::KAN* | This study |
| yHG54 | *ura3 ade2-1 leu2,3-112 his3-11,15 trp1Δ yhh1Δ::TRP1* (*yhh1-3* leu2 CEN) *rad7Δ::KAN* | This study |
| yHG104-6B | *MAT**a** ura3-1 ade2-1 leu2-3,112 trp1-1 his3-11,15 rat1-1* | This study |
| yHG104-2C | *MAT**a** ura3-1 ade2-1 leu2-3,112 trp1-1 his3-11,15 rat1-1 rad7Δ::KAN* | This study |
| MGSC102 | *MATa ura3-1 trp1-1 ade2-1 leu2-3,112 his3-11,15 rad26Δ::HIS3* | [4] |
| R267-10A | *MATa ura3-1 trp1-1 ade2-1 leu2-3,112 his3-11,15 rad26Δ::HIS3 rad7Δ::URA3* | [5] |
| yHG86-1A | *MATa ura3-1 ade2-1 leu2-3,112 trp1-1 his3-11,15 rad26Δ::HIS3 rna14-1* | This study |
| yHG86-3C | *MATa ura3-1 ade2-1 leu2-3,112 trp1-1 his3-11,15 rad26Δ::HIS3 rad7Δ::URA3 rna14-1* | This study |
| yHG87-1D | *MATa ura3-1 ade2-1 leu2-3,112 trp1-1 his3-11,15 rad26Δ::HIS3 hrp1-5* | This study |
| yHG87-1A | *MATa ura3-1 ade2-1 leu2-3,112 trp1-1 his3-11,15 rad26Δ::HIS3 rad7Δ::URA3 hrp1-5* | This study |
| yHG106-1D | *MAT**a** ura3-1 ade2-1 leu2-3,112 trp1-1 his3-11,15 rad52Δ::KAN* | This study |
| yHG106-6B | *MAT**a** ura3-1 ade2-1 leu2-3,112 trp1-1 his3-11,15 rad52Δ::KAN rna14-1* | This study |
| yHG112-7A | *MAT**a** ura3-1 ade2-1 leu2-3,112 trp1-1 his3-11,15 Ku70Δ::HYGR* | This study |
| yHG112-2A | *MAT**a** ura3-1 ade2-1 leu2-3,112 trp1-1 his3-11,15 Ku70Δ::HYGR rna14-1* | This study |
| yHG113-11A | *MAT**a** ura3-1 leu2-3,112 trp1-1 his3-11,15 lys2Δ ogg1Δ::KAN ntg1Δ::KAN ntg2Δ::KAN* | This study |

| | | |
|---|---|---|
| yHG113-11B | *MATa ura3-1 ade2-1 leu2-3,112 trp1-1 his3-11,15 met15Δ ogg1Δ::KAN ntg1Δ::KAN ntg2Δ::KAN rna14-1* | This study |
| yHG115-6A | *MATa ura3-1 ade2-1 leu2-3,112 trp1-1 his3-11,15 sml1Δ::URA3* | This study |
| yHG115-1B | *MATa ura3-1 ade2-1 leu2-3,112 trp1-1 his3-11,15 sml1Δ::URA3 rna14-1* | This study |
| yHG115-8A | *MATa ura3-1 ade2-1 leu2-3,112 trp1-1 his3-11,15 sml1Δ::URA3 mec1Δ::LEU2* | This study |
| yHG115-3C | *MATa ura3-1 ade2-1 leu2-3,112 trp1-1 his3-11,15 sml1Δ::URA3 mec1Δ::LEU2 rna14-1* | This study |
| yHG116-4C | *MATa ura3-1 leu2-3,112 trp1-1 his3-11,15 msh2Δ::KAN* | This study |
| yHG116-13A | *MATa ura3-1 leu2-3,112 trp1-1 his3-11,15 msh2Δ::KAN rna14-1* | This study |
| yHG118-8D | *MATa ura3-1 ade2-1 leu2-3,112 trp1-1 his3-11,15 rad1Δ::LEU2* | This study |
| yHG118-7D | *MATa ura3-1 ade2-1 leu2-3,112 trp1-1 his3-11,15 rad1Δ::LEU2 rna14-1* | This study |
| yHG119-3C | *MATa ura3-1 ade2-1 leu2-3,112 trp1-1 his3-11,15 rad9Δ::KAN* | This study |
| yHG119-4C | *MATa ura3-1 leu2-3,112 trp1-1 his3-11,15 rad9Δ::KAN rna14-1* | This study |
| yHG120-8B | *MATa ura3-1 ade2-1 leu2-3,112 his3-11,15 rad18Δ::KAN* | This study |
| yHG120-2C | *MATa ura3-1 leu2-3,112 trp1-1 his3-11,15 rad18Δ::KAN rna14-1* | This study |
| yHG121-11C | *MATa ura3-1 ade2-1 leu2-3,112 trp1-1 his3-11,15 lig4Δ::KAN* | This study |
| yHG121-4A | *MATa ura3-1 ade2-1 leu2-3,112 trp1-1 his3-11,15 lig4Δ::KAN rna14-1* | This study |
| yHG124-7A | *MATa ura3-1 ade2-1 leu2-3,112 trp1-1 his3-11,15 bar1Δ* | This study |
| yHG124-6D | *MATa ura3-1 ade2-1 leu2-3,112 trp1-1 his3-11,15 bar1Δ rna14-1* | This study |
| yHG136-6A | *MATa ura3-1 ade2-1 leu2-3,112 trp1-1 his3-11,15 bar1Δ sic1Δ::KAN* | This study |
| yHG136-17B | *MATa ura3-1 ade2-1 leu2-3,112 trp1-1 his3-11,15 bar1Δ sic1Δ::KAN rna14-1* | This study |
| yHG144-5D | *MATa ura3 ade2-1 leu2-3,112 trp1-1 his3-11,15 bar1Δ::HYGR ura3::URA3/GPD-TK(7x)* | This study |
| yHG147-1D | *MATa ura3 ade2-1 leu2-3,112 trp1-1 his3-11,15 bar1Δ rna14-1 ura3::URA3/GPD-TK(7x)* | This study |
| yHG46-15C | *MATa ura3-1 ade2-1 leu2-3,112 trp1-1 his3-11,15 def1Δ::HYGR* | [6] |
| yHG95-10D | *MATa ura3-1 ade2-1 leu2-3,112 trp1-1 his3-11,15 def1Δ::HYGR rna14-1* | This study |
| yHG97-7B | *MATa ura3-1 ade2-1 leu2-3,112 trp1-1 his3-11,15 def1Δ::HYGR rna15-1* | This study |
| yHG96-6A | *MATa ura3-1 ade2-1 leu2-3,112 trp1-1 his3-11,15 def1Δ::HYGR hrp1-5* | This study |
| yHG104-13A | *MATa ura3-1 ade2-1 leu2-3,112 trp1-1 his3-11,15 def1Δ::HYGR rat1-1* | This study |


1. Amrani N, Minet M, Wyers F, Dufour ME, Aggerbeck LP, et al. (1997) PCF11 encodes a third protein component of yeast cleavage and polyadenylation factor I. Mol Cell Biol 17: 1102-1109.
2. Dichtl B, Blank D, Sadowski M, Hubner W, Weiser S, et al. (2002) Yhh1p/Cft1p directly links poly(A) site recognition and RNA polymerase II transcription termination. EMBO J 21: 4125-4135.
3. Verhage R, Zeeman AM, de Groot N, Gleig F, Bang DD, et al. (1994) The RAD7 and RAD16 genes, which are essential for pyrimidine dimer removal from the silent mating type loci, are also required for repair of the nontranscribed strand of an active gene in Saccharomyces cerevisiae. Mol Cell Biol 14: 6135-6142.

4. van Gool AJ, Verhage R, Swagemakers SM, van de Putte P, Brouwer J, et al. (1994) RAD26, the functional S. cerevisiae homolog of the Cockayne syndrome B gene ERCC6. EMBO J 13: 5361-5369.
5. Gonzalez-Barrera S, Prado F, Verhage R, Brouwer J, Aguilera A (2002) Defective nucleotide excision repair in yeast hpr1 and tho2 mutants. Nucleic Acids Res 30: 2193-2201.
6. Gaillard H, Wellinger RE, Aguilera A (2007) A new connection of mRNP biogenesis and export with transcription-coupled repair. Nucleic Acids Res 35: 3893-3906.
