# Supplementary material for: Cleavage Factor I Links Transcription Termination to DNA Damage Response and Genome Integrity Maintenance in Saccharomyces cerevisiae
Source: PLoS Genet. 2014 Mar 6;10(3):e1004203. doi: 10.1371/journal.pgen.1004203 (PMC3945788; doi:10.1371/journal.pgen.1004203)
Supplement: Table S4 — Primers used in this study. (PDF) [file pgen.1004203.s012.pdf]

Table S4. Primers used in this study

Name	Sequence
RPB2-A	5'-TCTTGGAATAATAACTTCGCGGC-3'
RPB2-B	5'-GGTGGATGACAAGATACATGCC-3'
ARS508 1-A	5'-CCCGTGGTAAACCTTTAGAAAAAC-3'
ARS508 1-B	5'-ATATGAACGGCAAATTGAGACAAA-3'
ARS508 2-A	5'-AGTCATTAATAGCAAAGCCGTACGT-3'
ARS508 2-B	5'-GGTCCTTTGATGTAACGATCATATTG-3'
ARS305 1-A	5'-TTCTTCCTCAGGTTTGCCTAGTG-3'
ARS305 1-B	5'-TGCCAAGTCTGATCGAAAAGC-3'
ARS305 2-A	5'-TGCTCTTAGTGCAAAACACGAAA-3'
ARS305 2-B	5'-TCCTCCGTGGTCGATTGTG-3'
ARS416 1-A	5'-CAGGCGCATACGCTACAATG-3'
ARS416 1-B	5'-GGTTGTTTGCAAGACCGAGAA-3'
ARS416 2-A	5'-TGTTGTTGTGCTGCTTGTTCTTT-3'
ARS416 2-B	5'-ATCCGCGCCGCAATT-3'
242210-242280 ChrV A	5'-TGCCTGCACGCCATTGT-3'
242210-242280 ChrV B	5'-TTCCCCACGGAAAGTTGTATCT-3'
